# Surgical Treatment of Congenital Facial Palsy: A Retrospective Analysis of More than 20 Years’ Experience in Smile Surgery

**DOI:** 10.3390/medicina62102039

**Published:** 2026-10-21

**Authors:** Andrea Ferri, Giorgia Menapace, Cecilia Zannoni, Laura Salvatori, Francesco Laganà, Silvano Ferrari, Bernardo Bianchi

**Affiliations:** 1 Maxillo-Facial Surgery Unit, Head and Neck Department, University Hospital of Parma, 43126 Parma, Italy; aferri@ao.pr.it (A.F.); czannoni@ao.pr.it (C.Z.); silvano.ferrari@unipr.it (S.F.); 2 Maxillo-Facial Surgery Unit, Department of Specialized Surgery, IRCCS Policlinico San Martino, 16132 Genoa, Italybernardo.bianchi@hsanmartino.it (B.B.)

**Keywords:** congenital facial palsy, Moebius syndrome, smile surgery, gracilis free flap, facial reanimation

## Abstract

*Background and Objectives*: Congenital facial palsy, either isolated or associated with syndromic conditions such as Moebius syndrome, is a rare disorder that poses significant functional and psychological challenges for patients, families, and healthcare teams. Dedicated care pathways and a multidisciplinary approach are essential for optimal management. This study presents our experience of more than 20 years in smile surgery, highlighting clinical outcomes with an objective analysis of facial symmetry and movement as well as a review of our progressive improvements of surgical techniques and patient management over time. *Materials and Methods*: Patients diagnosed with congenital facial palsy who were referred to our institution for rehabilitative treatment and surgical management between January 2003 and December 2025 were retrospectively reviewed. In those with standardized preoperative and postoperative photographs, commissure excursion (CE) and commissure excursion deviation (CEdev) were measured with Emotrics and compared before and after surgery within three groups defined by the laterality of the palsy and of the reanimation. *Results*: Ninety-six patients were included, of whom 56.2% had a syndromic pattern. Facial reanimation was achieved by gracilis muscle transfer innervated by cross-facial nerve grafting (CFNG), supercharged CFNG, or masseteric nerve innervation, and only two major complications occurred. Objective analysis was possible in 61 patients (64%). In unilateral palsy, CEdev during smiling decreased from a median of 14.7 to 2.4 mm, with improvement in all 28 patients (*p* < 0.0001). Across the whole cohort, CE of the operated side increased by a median of 11.6 mm (95% CI 10.2–13.1) and dynamic excursion by 5.6 mm (4.5–7.0). *Conclusions*: Gracilis muscle transfer produced a large, objectively measurable gain in facial symmetry and movement. These outcomes reflect the progressive specialization of our institution and the refinement of surgical timing, flap inset and innervation source, together with structured facial rehabilitation, which should ideally begin before surgery and continue postoperatively.

## 1. Introduction

Congenital facial palsy is an extremely rare condition, with an estimated incidence ranging from 0.2 to 2/1000 live births for isolated forms, while syndromic cases such as Moebius syndrome occur in approximately 1:50,000–1:250,000 live births [1,2,3]. Moebius syndrome, which is characterized by deficits of the VI and VII cranial nerves, is the most known condition associated with congenital facial palsy even though isolated VII nerve deficits or atypical cases with different cranial nerve involvement may be encountered [3,4].

Multidisciplinary management of these cases is mandatory: pediatric neurologists and neonatologists (or other dedicated specialists depending on the setting) often provide initial clinical evaluation and explore the presence of other deficits or features which may be part of a broader syndromic pattern; speech therapists and physiotherapists begin swallowing rehabilitation and facial training to manage feeding issues; and ophthalmologists manage strabismus, lagophthalmos and other ocular conditions [2,5,6]. It is therefore suggested that these patients should be referred to highly specialized centers that can meet the various needs and challenges associated with this condition.

One of the main challenges following a diagnosis of congenital facial palsy is its social and psychological impact on both the child and the family. Families are often confronted with a complex condition and may lack adequate information, guidance, and access to appropriate resources. Valuable assistance can be provided by patient associations (in Italy, for example, the Italian Association for Moebius Syndrome), which offer support to families, build networks with specialists, and, in some cases, help coordinate multidisciplinary care. These organizations connect families facing similar challenges, enabling them to make informed decisions, develop realistic expectations, and benefit from shared experiences. They bring together patients, families and clinicians, often creating a unique collaborative network in which all those involved can address clinical, social, and emotional issues collectively.

The management of congenital facial palsy varies depending on the characteristics of each individual patient, based on the severity of the condition [1]. Selected patients with incomplete facial paralysis may benefit from facial rehabilitation using speech therapy or physiotherapy as standalone treatments. Although each case is unique, smile surgery is generally considered the mainstay of treatment when indicated; in such cases, the procedure typically consists of a free gracilis neuromuscular transfer, currently regarded as the gold-standard procedure worldwide [7,8,9,10]. However, several technical aspects should be considered when performing this type of surgery. Over the years, these have evolved through insights gained from the published experience of centers worldwide and from our institution’s learning curve.

The aim of this paper is to present our 20 years’ experience in smile surgery, highlighting clinical outcomes and the progressive refinement of surgical techniques and clinical management over time.

## 2. Materials and Methods

### 2.1. Population and Inclusion Criteria

Patients with diagnosis of congenital facial paralysis who were referred to our center for rehabilitative therapy and/or surgery between January 2003 and December 2025 at the Maxillo-facial Surgery Unit, Head and Neck Department, University Hospital of Parma, Italy, were reviewed in this retrospective observational study.

We enrolled patients with complete or incomplete, unilateral or bilateral congenital facial palsy who underwent smile surgery involving gracilis free-flap transfer. Patients with syndromic conditions and/or other anatomical or functional abnormalities, as well as patients with isolated congenital facial paralysis without associated syndromic features, were included in the study. We included both patients who underwent surgery in childhood and patients who received surgical treatment in adult age.

Ethical approval was obtained from the Ethics Board of the University Hospital of Parma, and all the enrolled patients provided written informed consent to participate in the study; in pediatric patients, parental consent and patient assent were obtained. Inclusion criteria were: unilateral or bilateral congenital facial palsy, complete or incomplete; smile surgery at any age; minimum 12-month follow-up; and signed informed consent. Patients who underwent facial rehabilitation alone, and therefore did not undergo smile surgery, were excluded from the study.

Approximately 300 patients with congenital facial palsy were referred to our center between 2003 and 2025. Ninety-six of these patients met the inclusion criteria and were enrolled.

### 2.2. Care Pathway—First Evaluation and Multidisciplinary Management

Pediatric patients were generally referred to our center via patient associations or by external specialists such as neonatologists, pediatricians, or neurologists. In our institution, patients were managed according to an integrated care pathway specifically developed for congenital facial paralysis. During the initial outpatient evaluation, a multidisciplinary assessment was performed, involving a surgeon with expertise in smile surgery and a speech-language therapist with expertise in facial rehabilitation. Patients were examined for other possible cranial nerve deficits, and residual facial nerve function was assessed. Most patients were referred to our intitution with an established diagnosis, made by the referring pediatric neurologist or geneticist. When a syndromic association was suspected and had not been previously investigated, a genetic consultation was requested. Imaging investigations were reviewed or performed to exclude organic or progressive causes of facial palsy; other specialist consultations were obtained when indicated. The integrated care pathway also included ophthalmologic and dental evaluations. Patients were referred from across the country and, in some cases, from abroad. Therefore, multidisciplinary evaluations were scheduled on the same day to minimize travel burden and facilitate access to care.

**Diagnostic criteria**. Moebius syndrome was diagnosed according to the criteria agreed at the First Scientific Conference on Moebius Syndrome, requiring congenital, unilateral or bilateral, non-progressive facial weakness (VII cranial nerve) together with limited abduction of one or both eyes (VI cranial nerve). Other syndromes were diagnosed according to currently accepted clinical criteria (i.e., Poland syndrome, on the basis of the absence or hypoplasia of the sternocostal head of the pectoralis major, with or without associated ipsilateral chest wall, breast or upper limb anomalies; CHARGE syndrome), cytogenetically (i.e., Turner syndrome—45,X karyotype or variants), or with molecular analysis, when available. Isolated congenital facial palsy was a diagnosis of exclusion, defined as congenital, non-progressive facial weakness in the absence of involvement of other cranial nerves, of dysmorphic or systemic features, and of any history of birth trauma or of an acquired cause.

Once the overall evaluation had been completed, a rehabilitative and/or surgical treatment plan was proposed based on patient’s age, comorbidities, and the severity of facial palsy. Facial rehabilitation was generally indicated in patients with incomplete facial paralysis to enhance residual facial function.

### 2.3. Care Pathway—Pre-Operative Evaluation

At 5–6 years of age, patients were considered for smile surgery when rehabilitation alone was not sufficient to achieve satisfactory, expressive movement. Assessment for gracilis transfer surgery was focused on the middle third of the face and was exclusively clinical, based on observation during both voluntary and spontaneous facial movements; no instrumental analysis was used. The residual activity of the muscles involved in smile production was evaluated, in particular the zygomaticus complex, the levator labii superioris and orbicularis oris. The criteria for surgery differed according to the pattern of palsy. In unilateral complete palsy, the absence of any voluntary or spontaneous elevation of the oral commissure on the affected side, in the presence of a normally functioning contralateral side, was considered a sufficient indication. In bilateral complete palsy, the indication was based on the absence of commissural movement on both sides rather than on asymmetry, which is minimal in these patients. In incomplete palsy, whether unilateral or bilateral, the indication was the most difficult to establish and was based on the combination of insufficient residual elevation of the commissure and a degree of asymmetry considered functionally and esthetically relevant during spontaneous smiling. In patients with bilateral incomplete palsy the two sides were rarely affected to the same degree. Bilateral reanimation was reserved for patients in whom neither side retained sufficient residual elevation of the commissure, whereas unilateral reanimation was performed when one side retained enough residual function to contribute to smiling, the more severely affected side alone being addressed surgically.

In all patients the decision to proceed with smile surgery was taken together with the patient and the family, after discussion of the expected benefit and of the alternatives.

Patients and their parents were informed about the objectives, expected benefits, and potential complications associated with the procedure. Written informed consent to surgery was obtained.

### 2.4. Care Pathway—Surgical Technique

According to the literature, patients with unilateral facial paralysis and a healthy contralateral facial nerve have undergone facial reanimation using a gracilis free muscle transfer innervated via a short cross-facial nerve graft (CFNG) from the contralateral facial nerve. In such cases, surgery included a first step stage for the CFNG, followed, 8–10 months later, by the assessment of axonal regeneration through the CFNG using Tinel’ sign. If Tinel’ sign was positive, patient underwent second-stage surgery consisting of gracilis muscle transfer.

Since its introduction in our practice in 2016, the supercharged CFNG technique has generally been preferred in adults, in order to avoid weak flap contraction. In adults, axonal regeneration along a cross-face nerve graft has been reported to be slower and less efficient, with a correspondingly longer denervation time before reinnervation of the transferred muscle, so that the axonal load delivered by a single graft may be insufficient [11]. On this basis, the masseteric nerve on the healthy side was used as an additional source of axonal input in adult patients to provide a more reliable and more rapidly available input to the flap. Supercharged CFNG was performed in younger patients with bilateral incomplete palsy, in whom weakness was also present on the less-affected side, and whenever concerns existed regarding the reliability of innervation, typically when the middle branches of the contralateral facial nerve were found to be of reduced caliber during intraoperatively assessment [12]. In this approach, the masseteric nerve on the healthy side was used as an additional source of axonal input to enhance graft reinnervation.

Gracilis reinnervation was achieved using the ipsilateral masseteric nerve when no healthy facial nerve was available as a donor—as in most patients with bilateral facial paralysis—or when the contralateral facial nerve, although present, was considered too compromised to be used without further weakening the residual function of the less-affected side.

In patients undergoing bilateral facial reanimation, the contralateral side was addressed only once satisfactory voluntary control of the previously operated side had been achieved, which was expected approximately 6 to 7 months after the first procedure: 3 to 4 months for activation of the gracilis innervated by the masseteric nerve, followed by 2 to 3 months of speech therapy and rehabilitation. The second transfer was therefore never performed earlier than 6 months after the first.

### 2.5. Care Pathway—Post-Operative Management and Follow-Up

Patients required a short hospital stay after CFNG procedure (2–3 days), whereas following gracilis free-flap transplantation hospitalization typically lasted 6–7 days. In these cases, a dedicated postoperative care protocol is followed to optimize patient recovery, including management of surgical drains and wounds, and the progressive restoration of oral intake and ambulation.

Approximately 30 days from the gracilis flap transfer, the facial rehabilitation program was initiated. The speech therapist began with scars and cheek massages to promote lymphatic drainage, reduce postoperative edema, and relax the transplanted muscle. Approximately 2 months after surgery, the patient was instructed to begin facial retraining aimed at achieving synchronous facial movements. Once the first muscle activation was observed (typically after 3–4 months), the training focused on improving the muscle contractile strength and motor control. Different rehabilitation strategies were adopted depending on the source of innervation; among these, neuromuscular retraining and the FIT-SAT [13] were the mainly used in our experience. Particularly, the FIT-SAT approach was developed in order to support the post-surgical rehabilitation in patients with congenital facial palsy when the innervation was provided by both contralateral masseteric and facial nerves. In these cases, gracilis flap movement was initially driven by the masseteric nerve and later by the contralateral facial nerve. Once activation via facial nerve (and thus spontaneous movement) began, the goal of facial rehabilitation focused on achieving separation of chewing and smiling.

### 2.6. Characteristics of Population, Surgery and Follow-Up

Data about sex (male vs female), kind of access pattern (by parent association vs autonomous), age at surgery, affected side (unilateral vs bilateral), severity of palsy (complete vs incomplete), gracilis source of reinnervation (masseteric nerve, CFNG, supercharged SCNG, or accessory nerve), CFNG to gracilis inter-stage interval (in cases of unilateral reanimation), interval between the two gracilis transfers (in bilateral reanimation), harvested gracilis dimension (half vs one-third of the muscle), failure, follow-up and other surgical procedures (including upper lip intervention and orthognathic surgery) were collected and analyzed.

Patients were divided into the following three groups according to the laterality of the palsy and of the reanimation: unilateral palsy with one side operated (Group A); bilateral palsy with one side operated (Group B); and bilateral palsy with both sides operated (Group C).

### 2.7. Clinical Assessment

Patients for whom standardized photographs were available both at baseline, prior to smile surgery, and at the most recent postoperative assessment, were included. The photos were obtained at least 12 months after gracilis transfer (after the second gracilis transfer, in patients with bilateral reanimation). Patients for whom preoperative or postoperative photographs were not available, or whose photographs did not meet the standardization criteria, were not included in this assessment. Photographs were obtained in a frontal view, at rest and during maximal voluntary smiling. Facial measurements were performed using Emotrics (Massachusetts Eye and Ear Infirmary, Boston, MA, USA), a validated machine learning-based tool for automated facial landmark placement and analysis in patients with facial palsy [14]. Measurements are returned in millimeters (mm). Automatically placed landmarks were reviewed and, where necessary, manually corrected by a single investigator. The following parameters were analyzed, at rest and during smiling: commissure excursion (CE) of each side; commissure excursion deviation (CEdev); and commissure height deviation (CHdev). CEdev and CHdev were computed as the absolute difference between the two sides, representing the magnitude of asymmetry, so that lower values indicate greater symmetry.

In patients who underwent revision surgery, the preoperative measurement refers to the assessment before the index procedure and the postoperative measurement to the assessment after the definitive procedure, so that the reported outcome reflects the complete reanimation pathway.

### 2.8. Data Synthesis and Statistical Analysis

Data were recorded and organized using Microsoft Excel spreadsheets (Microsoft Corporation, Redmond, WA, USA).

Continuous variables are reported as median (interquartile range) and, where indicated, as mean (standard deviation). No data were missing.

To assess the representativeness of the analyzed sample, patients included in the objective analysis were compared with those excluded, using the chi-square test—or Fisher’s exact test when any expected cell count was below five—for categorical variables, and the Mann–Whitney U test for continuous variables.

The distribution of the three study groups across the study period was compared with the chi-square test, using three periods defined a priori by the two datable changes in surgical technique (2003–2010, 2011–2015 and 2016–2025).

Preoperative and postoperative measurements were compared within each study group (Groups A, B and C) using the Wilcoxon signed-rank test for paired data. The magnitude of change was quantified by the Hodges–Lehmann estimator of the median within-patient difference (postoperative minus preoperative), with a 95% confidence interval derived from the Wilcoxon distribution, and the effect size by the matched-pair rank-biserial correlation. The Shapiro–Wilk test was used to inspect the distribution of the within-patient differences. *p* values were adjusted with the Holm–Bonferroni procedure.

The association between age at surgery and outcome was assessed with the Spearman rank correlation coefficient, with age analyzed as a continuous variable. Because the within-patient change depends strongly on the baseline value, the postoperative measurement was used as the outcome of primary interest, and the effect of age was additionally examined in a linear model adjusting for the preoperative value. Patients were also classified as operated in childhood (<18 years) or in adulthood (≥18 years) and compared with the Mann–Whitney U test; this dichotomy is reported alongside the continuous analysis, since dichotomizing a continuous variable reduces statistical power and can produce results that depend on the chosen threshold.

The effect of the reduction in harvested muscle mass was assessed by comparing patients operated before 2011 (half of the gracilis) with those operated from 2011 onwards (one-third) with the Mann–Whitney U test; the between-group difference was quantified by the Hodges–Lehmann estimator with a 95% confidence interval. The postoperative value was the outcome of primary interest, and the comparison was repeated in a linear model adjusting for the preoperative value and, in a further specification, for follow-up duration and type of neural input.

All tests were two-sided and *p* values below 0.05 were considered statistically sig-nificant. Analyses were performed in Python 3.11 using SciPy 1.17, statsmodels 0.15 and pandas 3.0.

## 3. Results

### 3.1. Characteristics of Population

A total of 96 patients (38 male, 58 female) were included in this retrospective study. Fifty-eight patients came to our attention through patient associations, while a further 38 were referred by other specialists. Mean age at surgery was 14.8 (range 5–49); in particular, 68 (70.8%) underwent smile surgery in childhood (mean 8.6, median eight), and 28 (29.2%) received surgical treatment in adult age (mean 30.1, median 31).

Mean postoperative follow-up was 112.3 months (median 107, range 12–241); 82 patients (85.4%) were followed for at least 24 months and 42 (43.8%) for at least 120 months. Follow-up by group was 109 months (IQR 42 to 157) in Group A, 82 months (31 to 196) in Group B and 120 months (77 to 169) in Group C.

Forty-five had unilateral facial palsy, of whom 20 had an incomplete and 25 a complete pattern. Among the 51 patients with bilateral facial palsy, 29 had incomplete bilateral involvement and 22 had complete bilateral facial palsy. Fifty-four (56.2%) of the 96 enrolled patients were affected by a syndromic condition that also included facial paralysis or presented other anatomical or functional abnormalities (Moebius syndrome, 51%; Moebius with Poland syndrome 2.1%; Moebius with Turner syndrome 1%; and CHARGE syndrome 2.1%). Forty-two patients (43.8%) had isolated congenital facial paralysis without associated syndromic or additional features (Table 1).

### 3.2. Characteristics of Surgery

Data were summarized in Table 1. Forty-five patients had unilateral palsy with one side operated (Group A), 25 with complete and 20 with incomplete unilateral facial palsy; 22 had bilateral palsy with one side operated (Group B), and all of them had incomplete palsy; and 29 had bilateral palsy with both sides operated (Group C), the majority (22 patients) with complete bilateral palsy and the remaining seven with incomplete palsy.

In patients undergoing a two-stage reconstruction, the median interval between the CFNG and the gracilis transfer was 10 months (IQR 9 to 12; *n* = 57). The interval was one month longer in patients operated in adulthood than in childhood (median 11 versus 10 months; *p* = 0.036). In patients undergoing bilateral reanimation, the median interval between the two gracilis transfers was 10 months (IQR 9 to 11; *n* = 29), with no difference between the two age groups (median 10 versus 10 months; *p* = 0.656). These intervals reflect not only the biological time required for reinnervation but also a variable organizational delay.

#### 3.2.1. Evolution of the Surgical Practice over the Study Period

**Figure 1:**
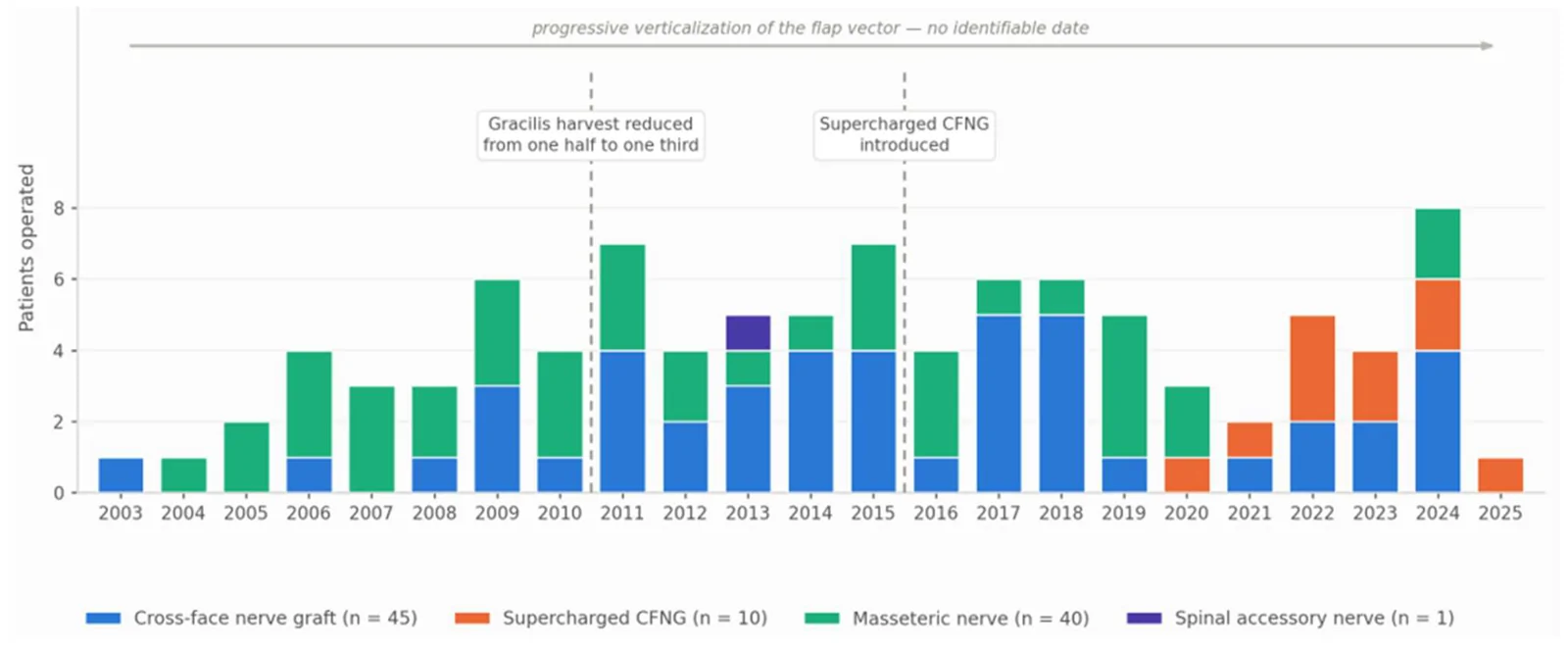
Patients enrolled in the study by year of surgery and source of innervation, 2003–2025. Bars are stacked by the source of innervation of the definitive procedure. The vertical dashed lines mark the two datable changes in surgical technique: the reduction in the gracilis harvest from one half to one third of the muscle in 2011, and the introduction of the supercharged cross-face nerve graft in 2016. The progressive verticalization of the flap vector occurred gradually over the study period and cannot be assigned to a specific year. The figure describes the patients enrolled in the study, and not the overall surgical activity of the unit. CFNG, cross-face nerve graft.

Patients were operated at a median rate of four per year (range 1 to 8) throughout the study period, with a reduction in 2020 and 2021 (three and two patients, respectively) that likely reflects the impact of the COVID-19 pandemic on elective surgical activity. The source of innervation changed over time in a way that differed between groups (Figure 1). In patients with unilateral palsy (Group A), the masseteric nerve was used in 4 of the 9 patients operated up to 2010 and in none thereafter, the cross-face nerve graft becoming the standard and being supplemented by the super-charged technique in the most recent years. A similar shift was observed in patients with bilateral palsy and one side reanimated (Group B), in whom the masseteric nerve accounted for 5 of the 7 patients operated up to 2010 and was subsequently used in only two patients, both with severe bilateral involvement. In patients with both sides reanimated (Group C), the masseteric nerve was the only source used throughout the study period, as no alternative was available. The distribution of the three groups did not differ significantly across the study period (*p* = 0.40), indicating that these changes reflect a change in surgical choice rather than a change in the case mix.

The following two further modifications of the technique are marked on the figure: the reduction in the gracilis harvest from one half to one third of the muscle in 2011, and the introduction of the supercharged cross-face nerve graft in 2016. These observations describe the evolution of our institutional practice.

#### 3.2.2. Group A

Forty-five patients had unilateral facial palsy with one side operated, 25 with complete and 20 with incomplete unilateral palsy.

**Figure 2:**
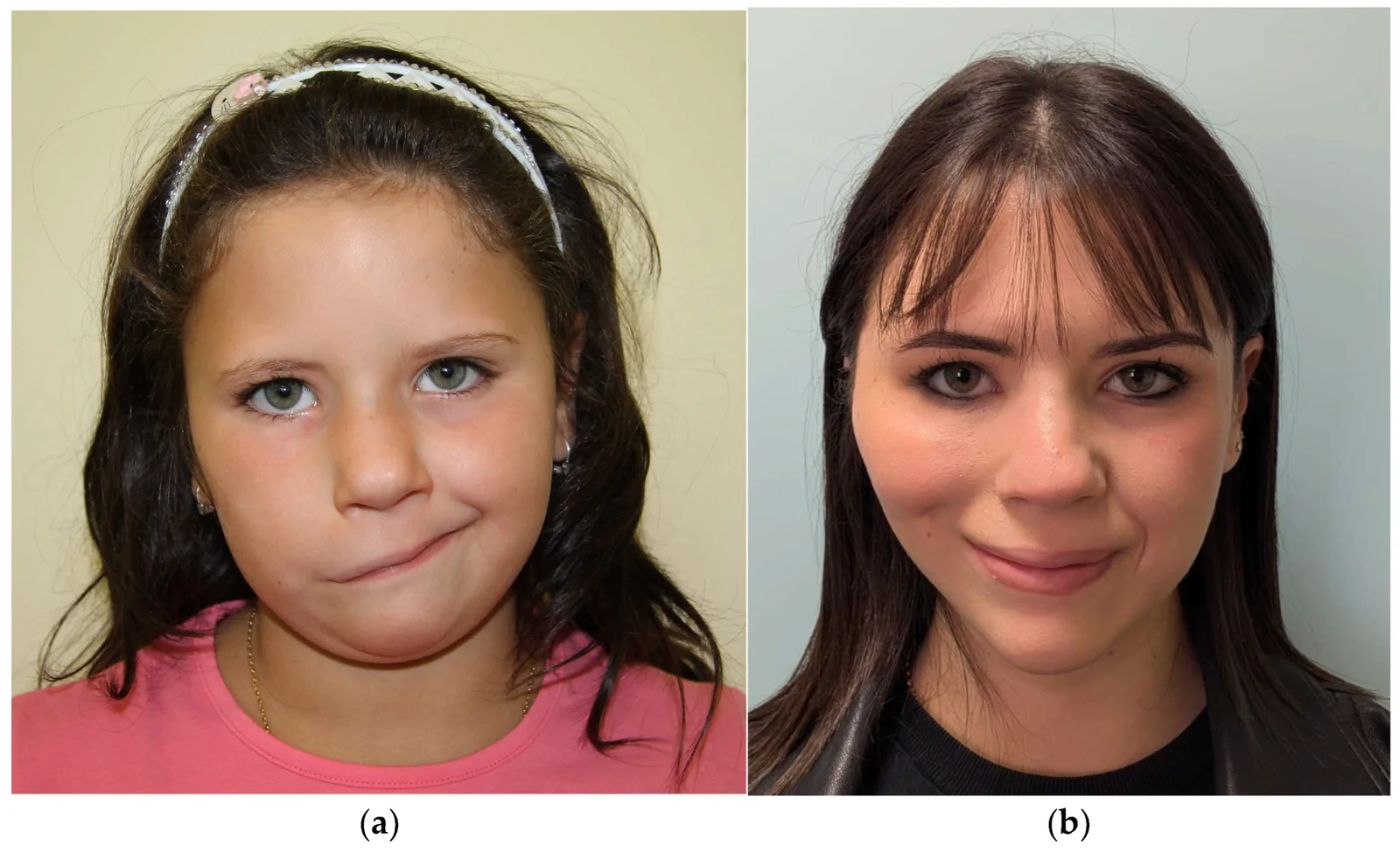
(**a**) Preoperative photograph of a 6-year-old patient with unilateral facial paralysis while smiling; (**b**) long-term postoperative photograph of the patient, 15 years after facial reanimation with gracilis muscle transfer, CFNG innervation.

**Figure 3:**
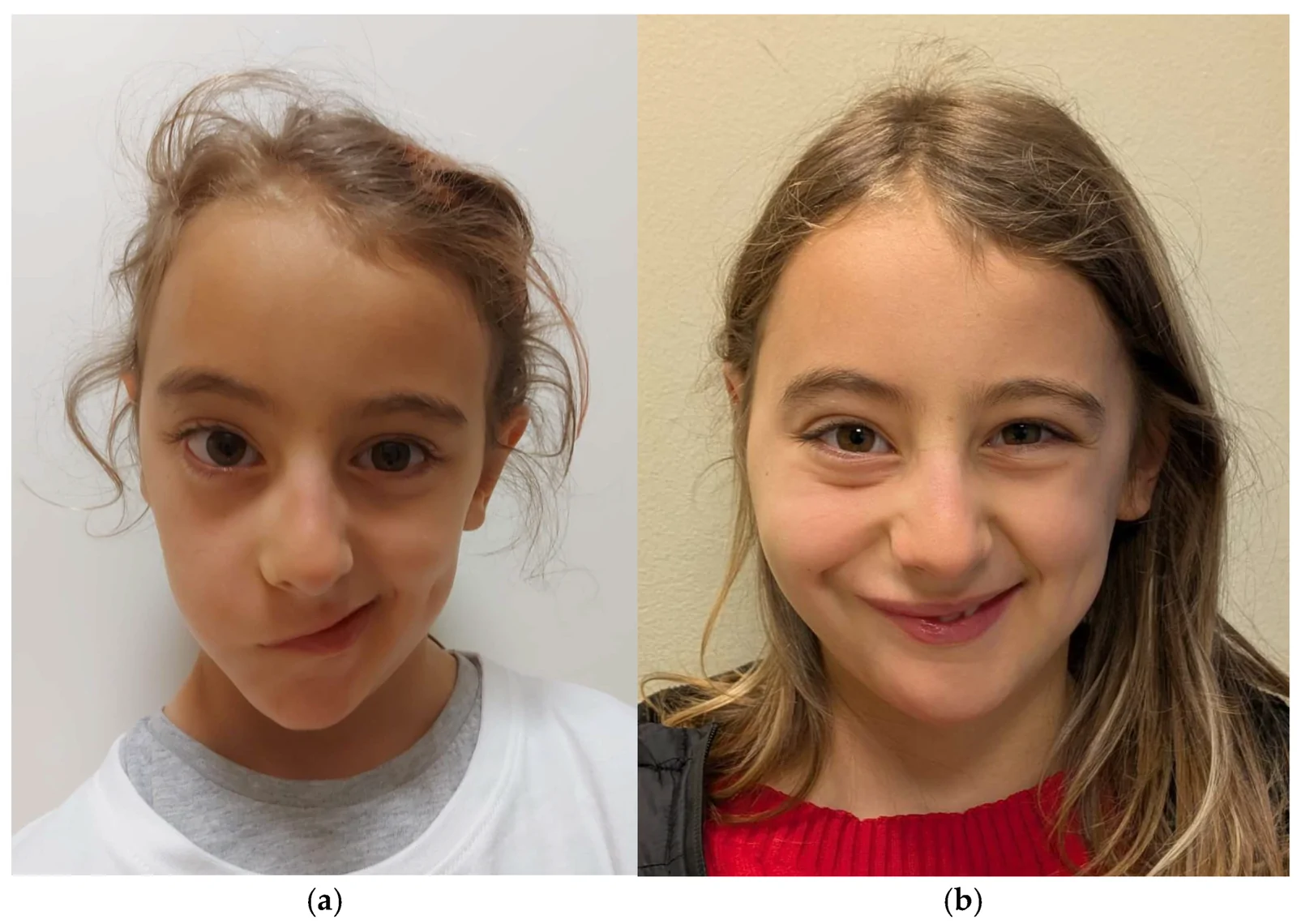
(**a**) Preoperative photograph of a 8-year-old patient with unilateral facial paralysis while smiling; (**b**) postoperative photograph of the patient, 1 year after facial reanimation with gracilis muscle transfer, CFNG innervation.

**Figure 4:**
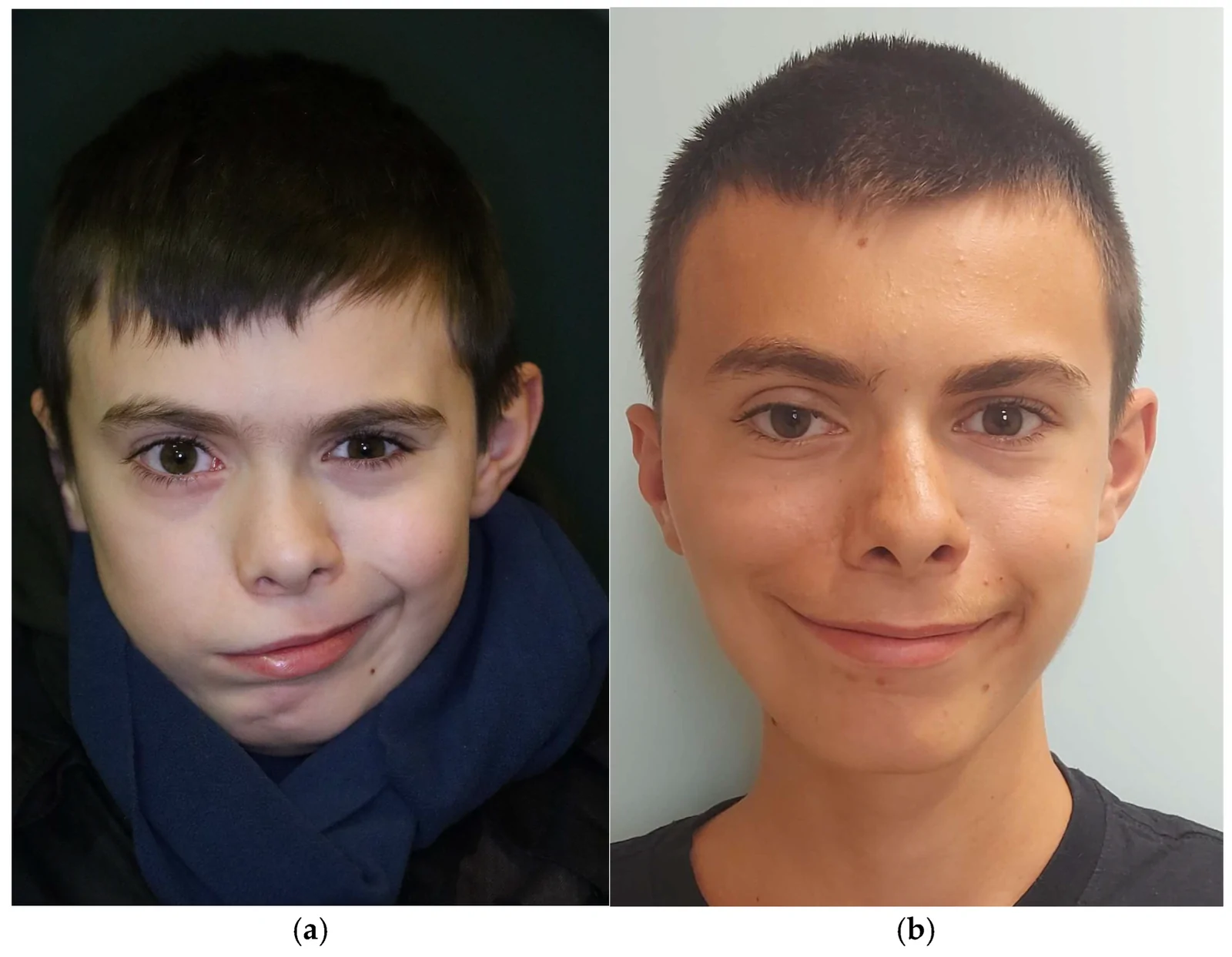
(**a**) Preoperative photograph of a 10-year-old patient with unilateral facial paralysis while smiling; (**b**) postoperative photograph of the patient, 5 years after facial reanimation with gracilis muscle transfer, supercharged CFNG innervation.

Nineteen patients underwent CFNG followed by gracilis muscle transfer (Figure 2a,b and Figure 3a,b). Three patients—two adults and one child—underwent a supercharged CFNG procedure (Figure 4a,b). Although the technique was introduced in our practice in 2016, the first patients treated with a supercharged cross-face nerve graft who met the inclusion criteria of the present study, including signed informed consent, were operated on from 2020 onwards.

Three patients received masseteric nerve innervation as follows: two as a single-stage gracilis muscle transfer, of whom one had previously undergone an unsuccessful CFNG-driven gracilis transfer performed abroad and the other was among the first cases of congenital facial palsy operated on at our institution, and one adult who achieved suboptimal contraction after CFNG-driven reinnervation and required revision surgery, consisting of gracilis repositioning and conversion to masseteric nerve innervation.

**Figure 5:**
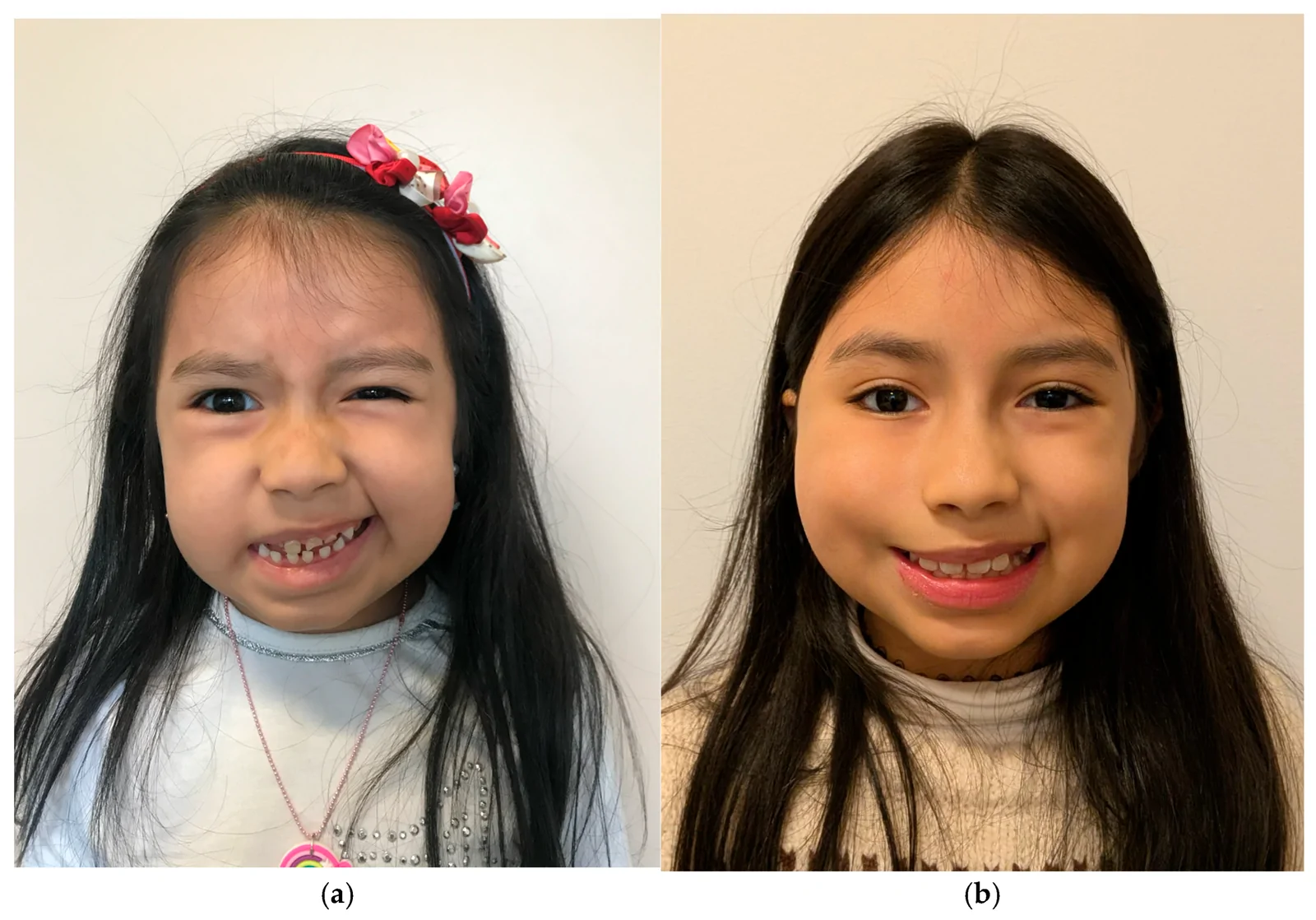
(**a**) Preoperative photograph of a 6-year-old patient with unilateral facial paralysis while smiling; (**b**) postoperative photograph of the patient, 1 year after facial reanimation with gracilis muscle transfer, CFNG innervation.

Sixteen patients with incomplete unilateral palsy (*n* = 20) underwent CFNG followed by gracilis muscle transfer (Figure 5a,b). Two patients underwent a supercharged CFNG. The remaining two patients (adults) underwent gracilis muscle transfer using the masseteric nerve (*n* = 1, one of the first patients with congenital facial palsy operated on at our institution) or the spinal accessory nerve (*n* = 1, no availability of ipsilateral masseteric nerve) as the donor nerve.

#### 3.2.3. Group B

**Figure 6:**
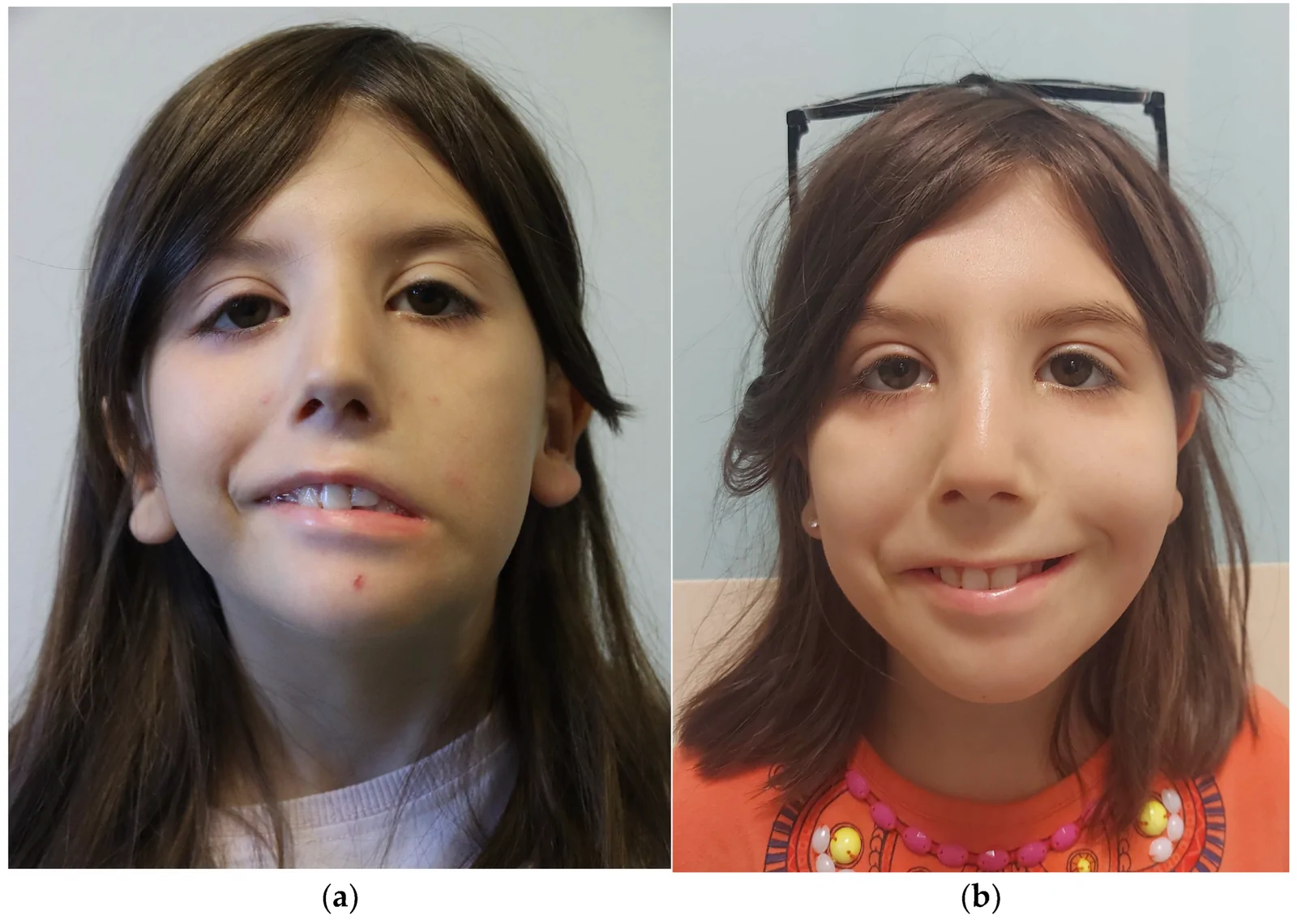
(**a**) Preoperative photograph of a 7-year-old patient with bilateral incomplete facial paralysis while smiling; (**b**) postoperative photograph of the patient, 4 years after unilateral facial reanimation with gracilis muscle transfer, supercharged CFNG innervation.

Ten patients underwent CFNG and five a supercharged CFNG procedure (Figure 6a,b).

Seven patients received masseteric nerve innervation, all with Moebius syndrome: five were operated between 2004 and 2008, before the introduction of the supercharged technique, and two more recently, in 2019 and 2024. In these two patients the palsy was severe on both sides, and the masseteric nerve was preferred in order to avoid harvesting axons from the contralateral facial nerve and further weakening the residual function of the less-affected side.

#### 3.2.4. Group C

Twenty-nine patients had bilateral palsy with both sides operated.

Seven patients had incomplete bilateral and 22 had complete bilateral facial palsy.

All had Moebius syndrome.

**Figure 7:**
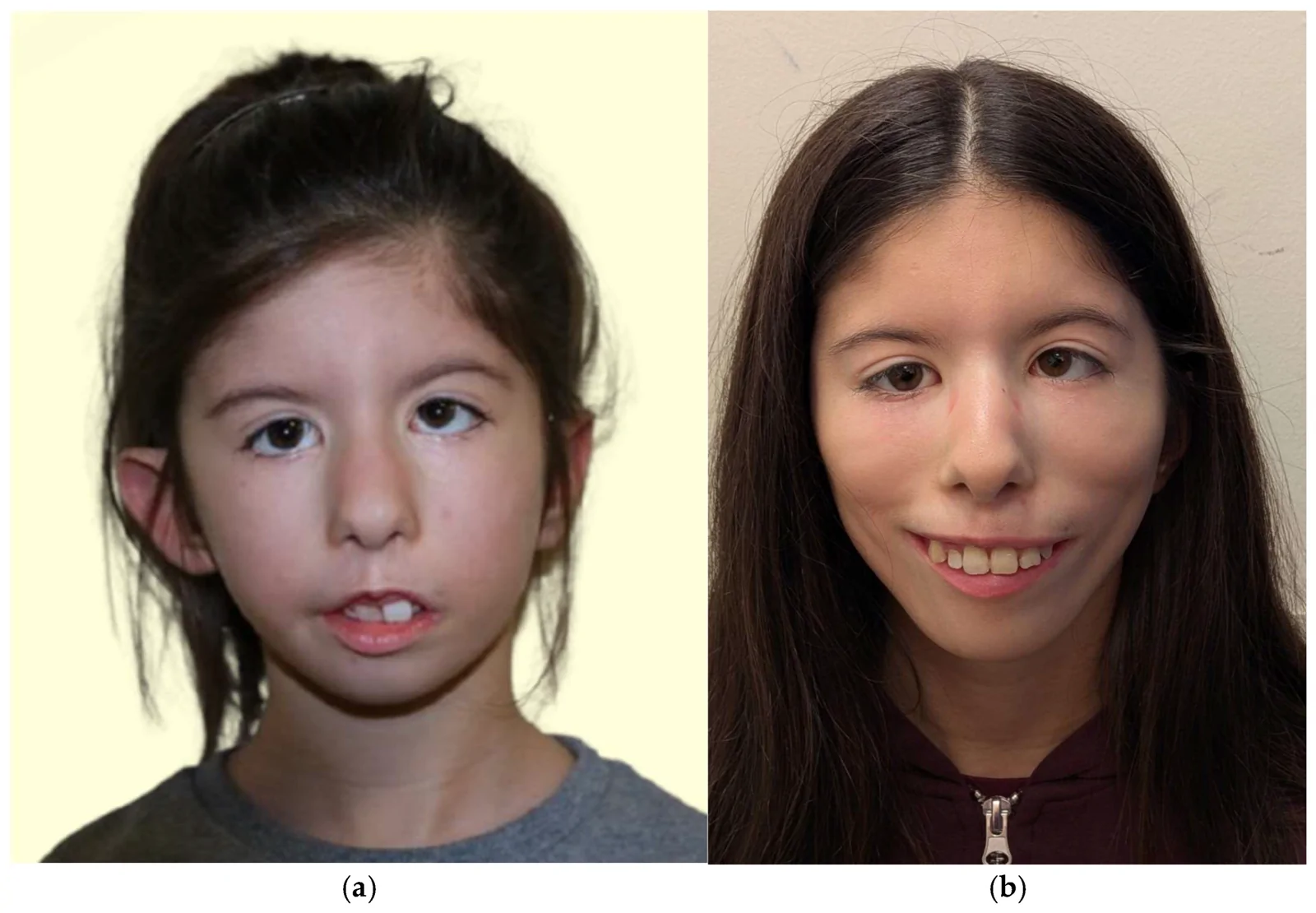
(**a**) Preoperative photograph of a 6-year-old patient with bilateral facial paralysis while smiling; (**b**) postoperative photograph of the patient, 8 years after bilateral facial reanimation with gracilis muscle transfer, masseteric innervation.

All received bilateral gracilis muscle transfers innervated by the ipsilateral masseteric nerves (Figure 7a,b).

**Figure 8:**
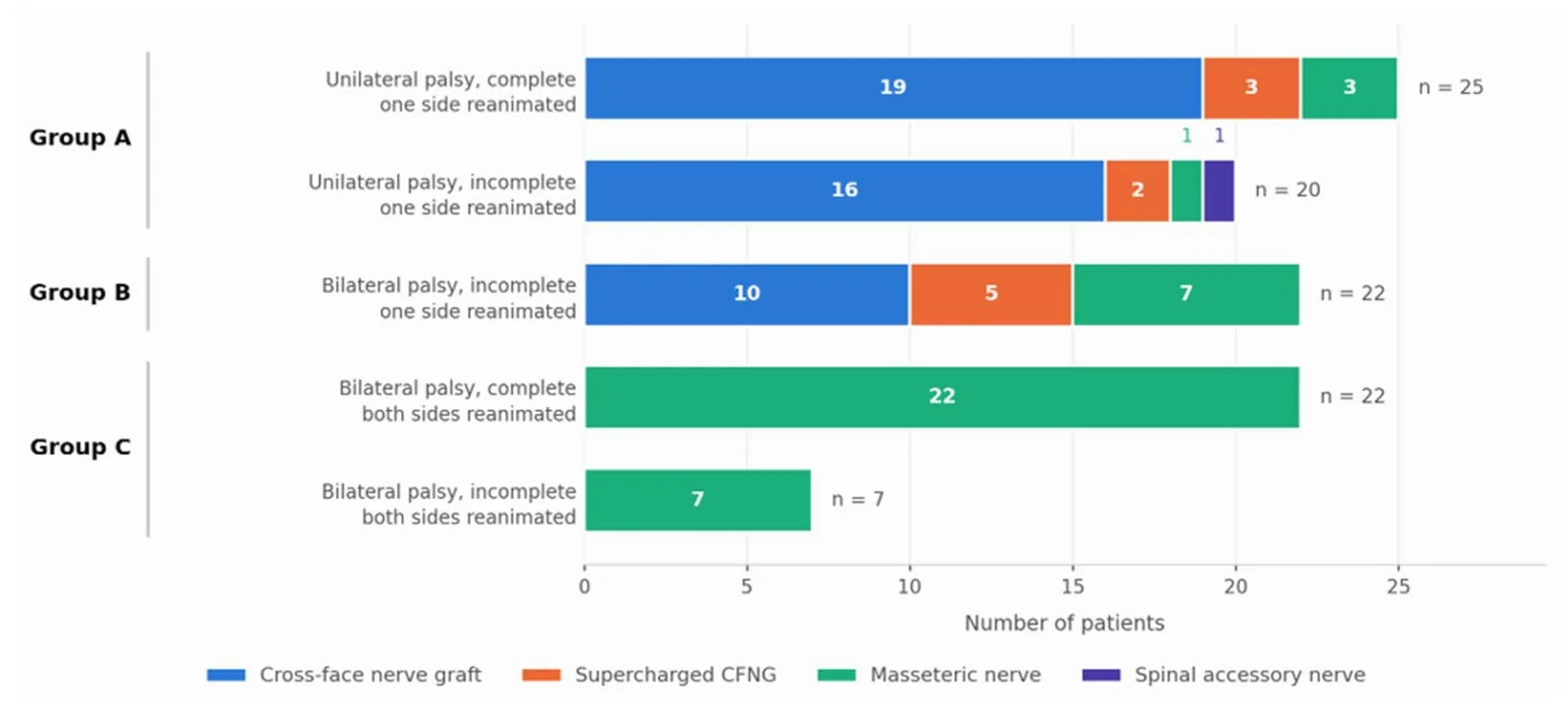
Source of innervation according to the pattern of facial palsy and the study group (96 patients). Group A, unilateral palsy with one side reanimated; Group B, bilateral palsy with one side reanimated; Group C, bilateral palsy with both sides reanimated. Numbers within the bars are patients, classified according to the source of innervation of the definitive procedure. CFNG, cross-face nerve graft.

Source of innervation by pattern of palsy and study group was reported in Figure 8.

### 3.3. Surgical Outcome

Major complications included failure of contralateral facial nerve-mediated gracilis muscle reinnervation in one case, which required surgical revision with a secondary gracilis muscle transfer reinnervated by the ipsilateral masseteric nerve. In one adult patient, suboptimal but present contraction was achieved after gracilis muscle transfer reinnervated via cross-grafting without a supercharged procedure. Revision surgery consisted of gracilis muscle repositioning and conversion to masseteric nerve innervation, with subsequent improvement in results.

Minor complications included: hematoma of the donor thigh site (two patients) and recipient cheek site (two); infection of the recipient site (three patients). Transient post-operative weakness of the operated thigh was observed in two patients following dissection of the nerve branch for gracilis muscle near the obturator foramen. No patient required re-exploration for flap ischemia. Esthetic sequelae included unsatisfactory scar appearance in six cases.

### 3.4. Other Surgical Procedures and Refinements

Fifteen patients (15.6%) underwent an upper lip procedure to correct lip atrophy; all had Moebius syndrome. Thirteen received lipofilling of the upper lip, and in two of them lip elongation was also performed. The remaining two chose hyaluronic acid filler for lip volumization.

Five patients (Moebius syndrome patients) had skeletal abnormalities—micrognathia; three patients (3.1%) underwent orthognathic surgery, while two patients (2.1%) underwent isolated genioplasty.

### 3.5. Clinical Assessment

Of the 96 patients who underwent gracilis free-flap transfer during the study period, 61 (63.5%) had standardized preoperative and postoperative photographs suitable for objective analysis; in the remaining 35, preoperative or postoperative photographs were either unavailable or did not meet the standardization criteria.

Among the 61 included patients, 28 were in Group A, 17 in Group B and 16 in Group C. Median age at surgery was 8 years (mean 13.2, range 5–48) and median postoperative follow-up was 102 months (mean 111.6, range 12–241).

Median postoperative follow-up of all the sixty-one patients was 102 months (IQR 36 to 179, range 12 to 241); 49 patients (80%) were assessed at least 24 months after surgery, and 42 (69%) at least 60 months and 28 (46%) at least 120 months.

Baseline demographic and clinical characteristics of the 61 patients are reported in Table 2.

Included and excluded patients did not differ significantly with respect to sex, age at surgery, palsy laterality, palsy grade, etiology, year of surgery, operated side, duration of follow-up, or the neural sources used for reinnervation (all *p* > 0.05, Table S1). The two largest differences were in age at surgery, which tended to be lower among included patients (median 8 years, range 5–48, versus 12 years, and range 6–49; *p* = 0.062), and in the use of the supercharged CFNG (9/61 versus 1/35; *p* = 0.087); neither reached statistical sig-nificance. Notably, the year of surgery did not differ between the two groups (median 2016 versus 2014; *p* = 0.512), indicating that the unavailability of standardized photographs was distributed across the whole study period rather than concentrated in the earlier years.

#### 3.5.1. Objective Measurements

**Figure 9:**
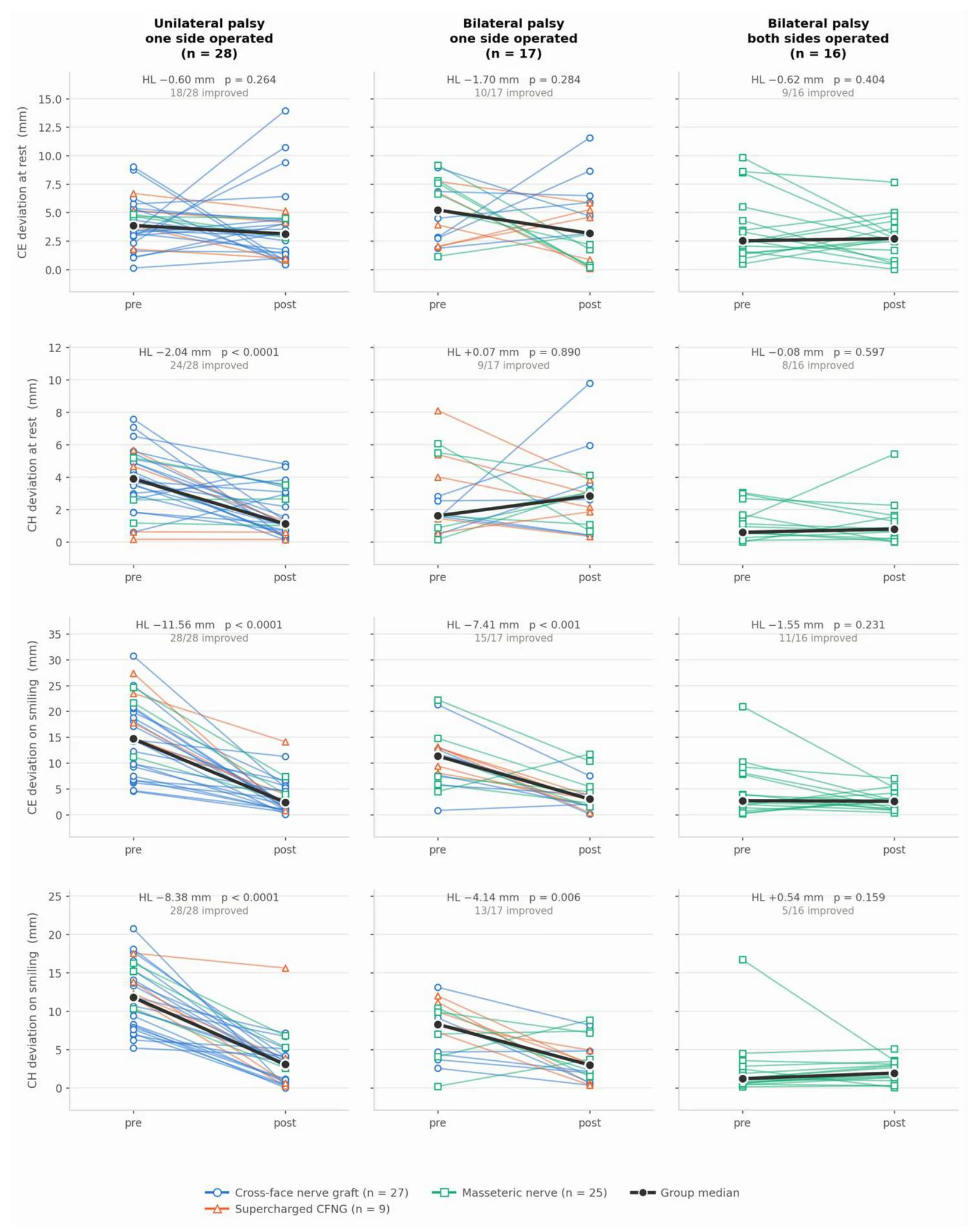
Facial symmetry before and after reanimation. Absolute right-to-left deviation of commissure excursion (CEdev) and commissure height (CHdev), at rest and on smiling, in the three study groups. Each line represents one patient and is colored according to the neural source driving the flap; the thick line joins the group medians. HL, Hodges–Lehmann estimate of the median within-patient change; *p* values from the exact Wilcoxon signed-rank test. Lower values indicate greater symmetry. For the patient who underwent revision, the neural source shown is that of the definitive procedure.

In patients with unilateral palsy (Group A), CE deviation on smiling decreased from a median of 14.7 mm (IQR 9.6–20.6) to 2.4 mm (1.0–4.8) and CH deviation on smiling from 11.8 mm (8.3–15.2) to 3.1 mm (1.0–4.4), corresponding to median within-patient reductions of 11.6 mm (95% CI 8.8 to 14.8) and 8.4 mm (6.8 to 10.2); all 28 patients improved on both measures (*p* < 0.0001). CH deviation at rest also improved (from 3.9 to 1.1 mm; reduction 2.0 mm, 1.1 to 3.0; and *p* < 0.001), whereas CE deviation at rest did not change (*p* = 0.26). The same pattern of smaller magnitude was observed in Group B, where CE and CH deviation on smiling decreased by 7.4 mm (4.1 to 9.9; *p* = 0.002) and 4.1 mm (1.6 to 6.2; *p* = 0.017) respectively, with no significant change at rest. In Group C no symmetry measure changed significantly (all *p* > 0.6), as expected: with both sides equally immobile before surgery these patients were already symmetric at baseline, so the right-to-left deviation is uninformative in this group (Figure 9, Table 3).

**Figure 10:**
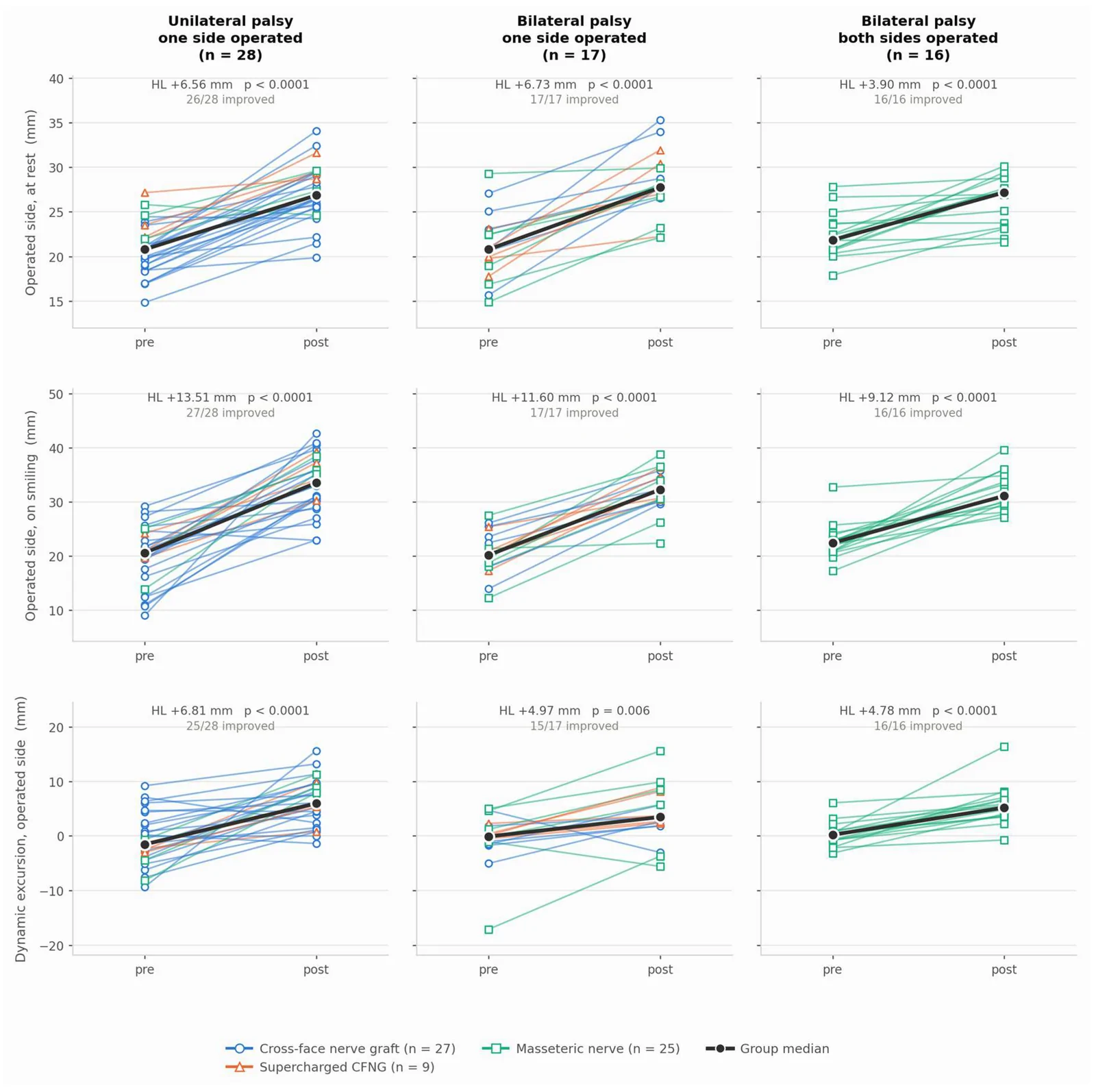
Commissure excursion of the operated side before and after reanimation. Dynamic excursion is the commissure position on smiling minus the position at rest on the same side and is a signed quantity, negative when the commissure is displaced towards the contralateral side on smiling. Each line represents one patient, colored by neural source; the thick line joins the group medians. HL, Hodges–Lehmann estimate; *p* values from the exact Wilcoxon signed-rank test.

Excursion of the operated side increased in all three groups (Figure 10, Table 3). Across the whole cohort, commissure excursion on smiling rose from 20.9 mm (19.3–23.8) to 32.3 mm (30.0–35.9), a median gain of 11.6 mm (10.2 to 13.1), and dynamic excursion from −0.2 mm (−2.1 to 1.8) to 5.5 mm (2.6–8.1), a gain of 5.6 mm (4.5 to 7.0), with 60/61 and 56/61 patients improving (both *p* < 0.0001). The gain in dynamic excursion was consistent across groups: 6.8 mm (4.6 to 9.1) in Group A, 5.0 mm (2.1 to 7.3) in Group B and 4.8 mm (3.3 to 6.8) in Group C, where all 16 patients improved. The negative preoperative values indicate that, before surgery, the affected commissure was displaced towards the contralateral side during smiling. Measurements of the non-operated side did not change in any of the 45 patients with a single operated side (all *p* > 0.08) (Figure S1, Table S2).

Descriptive statistics of all measurements before and after surgery, and the full output of the paired comparisons, are reported in Tables S3 and S4, respectively (Supplementary Materials).

#### 3.5.2. Neural Source

Postoperative measurements according to the neural source are reported in Table S5; the subgroups were too small, and too closely determined by the indication, to allow a formal comparison between neural sources.

#### 3.5.3. Age at Surgery

Across the whole cohort, the within-patient reduction in CE deviation on smiling was larger in older patients (rho = −0.26, *p* = 0.046; Figure S2a). This association reflected the baseline rather than the outcome: older patients presented with greater preoperative asymmetry (rho = +0.29, *p* = 0.025; Figure S2b), whereas the postoperative value was unrelated to age (rho = +0.03, *p* = 0.803; Figure S2c), and the effect of age was no longer apparent in a linear model adjusting for the preoperative measurement (*p* = 0.411). The same pattern, with stronger correlations, was observed in patients with unilateral palsy (Group A: change rho = −0.40, *p* = 0.036; preoperative value rho = +0.44, *p* = 0.018; post-operative value rho = −0.04, *p* = 0.825; and adjusted *p* = 0.464). In this study, age at surgery therefore influenced the degree of preoperative asymmetry and, consequently, the magnitude of the change achieved, but not the symmetry obtained after reanimation.

Patients operated in childhood (<18 years, *n* = 46) and in adulthood (*n* = 15) had a comparable duration of follow-up (98 versus 105 months, *p* = 0.750) and did not differ in any postoperative measurement: dynamic excursion of the operated side 5.7 versus 4.4 mm (*p* = 0.769), commissure excursion on smiling 31.7 versus 34.0 mm (*p* = 0.417), and CE deviation on smiling 2.5 versus 3.7 mm (*p* = 0.657).

#### 3.5.4. Duration of Follow-Up

Postoperative measurements did not vary with the duration of follow-up, either overall (dynamic excursion rho = 0.11, *p* = 0.42; CE deviation on smiling rho = 0.11, *p* = 0.39) or after adjustment for study group (*p* = 0.49). Since each patient contributed a single postoperative assessment and the duration of follow-up is almost entirely determined by the year of surgery (rho = −0.98), this is a comparison between patients assessed at different times rather than an assessment of stability within the same patient.

#### 3.5.5. Surgical Techniques and Changes

Since 2011 the amount of gracilis muscle harvested was reduced from one half to one third of the muscle. Nineteen patients were operated before 2011 and forty-two from 2011 onwards. Outcome did not differ between patients operated before and after the reduction in harvested muscle mass: in post-operative dynamic excursion of the operated side (median 5.7 mm vs 5.4 mm; Hodges–Lehmann difference 0.4 mm, 95% CI −2.1 to 3.1; and *p* = 0.680) nor in the pre- to post-operative change in excursion (1.8 mm, 95% CI −0.9 to 4.5; *p* = 0.222) (Figure S3).

## 4. Discussion

In this series, gracilis muscle transfer produced a large and objectively measurable gain in both symmetry and movement. The following discussion of our surgical practice should be read against this result: it describes how the procedure has been refined over more than two decades, not a demonstration that any single refinement improved the outcome.

When rehabilitation alone is not sufficient to achieve satisfactory, expressive movement, the patient is considered for smile surgery. A common question from families is when their child can undergo surgery. Despite the sense of urgency often expressed by parents, our experience suggests that the optimal age for surgery is approximately 5–6 years, depending also on the child’s overall psychophysical and developmental status [1,8,15]. This timing has been established over time based on factors such as self-perception, school integration with increasing social interaction, physical growth, and compliance during hospitalization. The latter is sometimes underestimated; however, particularly in free-flap procedures involving donor-site morbidity, drains, enforced bed rest, and intravenous therapy, patient cooperation plays a relevant role in preventing complications. In our objective analysis the symmetry achieved after reanimation was unrelated to age at surgery: older patients showed a larger change only because they presented with greater preoperative asymmetry. The choice of operating at 5 to 6 years therefore rests on developmental and psychosocial grounds rather than on a demonstrated functional advantage.

Regarding the most appropriate surgical technique for these patients, at the beginning of the century there was still ongoing debate, and pectoralis minor and latissimus dorsi neuromuscular flaps were among the options for facial reanimation [16,17,18,19]. Currently, the gracilis neuromuscular free flap is widely accepted as the gold standard procedure in facial reanimation worldwide [19,20,21,22], whereas alternative techniques are generally reserved for specific conditions, such as failure of gracilis transfer in both thighs (or of unilateral gracilis flap in patients with bilateral facial palsy), a depleted recipient vessel bed in the neck, or other rare clinical scenarios [15,16].

In our series of 96 patients who underwent gracilis neuromuscular transfer, no long-term donor-site morbidity was observed, and only two cases (2.1%) of failed contralateral facial nerve-driven gracilis flap reinnervation occurred, one required surgical revision with a secondary gracilis muscle transfer reinnervated by the ipsilateral masseteric nerve, and the other gracilis muscle repositioning and conversion to masseteric nerve innervation. Similar findings were observed when including patients with acquired facial palsy who underwent smile surgery but were not part of the present study, supporting the high reliability of the flap and its minimal morbidity.

Over the years, we have modified some technical aspects of the surgical procedure, thereby reducing the need for secondary refinements. One of these concerns the thickness of the gracilis flap harvested during the muscle splitting [23,24,25,26]. Initially, nearly half of the muscle circumference was harvested due to concerns regarding potential devascularization and denervation and, more importantly, progressive muscle atrophy occurring during the period of axonal regeneration prior to neuromuscular activation. This approach often led to an overestimation of flap volume, resulting in excessive bulk that occasionally required secondary debulking procedures. Currently, about one third of the muscle thickness is harvested—and in some patients slightly less—obtaining a muscle strip appropriately sized for the intended function. The objective outcome did not differ between patients operated before and after this change, indicating that the reduction in harvested muscle did not come at the cost of movement, although the comparison is confounded with surgical era and is not powered to establish equivalence.

Another important technical consideration is the choice of the flap vector. Initially, we positioned and secured the flap within a pre-auricular cheek pocket to avoid the bulky effect caused by overlap over the zygoma. However, in some cases this inset led to a “joker smile” deformity, resulting in an unpleasant smile shape and patient dissatisfaction. Currently, the flap vector has been verticalized and the flap is secured to the deep temporal fascia above the ear. Although this approach may result in some degree of flap overlap over the zygoma, a bulky cheek appearance is prevented by the previously described reduction in flap thickness. In addition, we no longer routinely remove the buccal fat pad, as was performed in the early years of facial animation surgery. The verticalization of the vector occurred progressively and could not be assigned to a specific year, and its effect was therefore not assessed in the present study.

Over time, we have modified the amount of tension applied to the gracilis flap during inset. Initially, flap length was estimated by measuring the distance between the oral commissure and the tragus and harvesting a flap 1–2 cm longer. This deliberate overestimation accounted for the space required for anchoring sutures at the oral commissure and fixation to the deep temporal fascia. The rationale was to better replicate physiological muscle contraction in its native environment in the thigh. This strategy proved only partially satisfactory: it was generally effective when gracilis flap innervation was driven by masseteric nerve, whereas muscle contraction was sometimes insufficient in patients with unilateral facial palsy when reinnervation was achieved via CFNG. Currently, we harvest a flap whose length matches the cheek pocket, applying slight overcorrection of tension. Intraoperatively, this results in a temporary forced smile, which progressively decreases and typically resolves within 3 weeks. When the gracilis flap is innervated by the masseteric nerve, lower tension is generally applied, taking into account the stronger contraction achieved during activation. This strategy, combined with the supercharged CFNG when indicated, has in our experience substantially reduced the occurrence of weak flap contraction; this observation reflects clinical impression and was not systematically recorded.

In cases of excessive contraction, facial rehabilitation therapy and botulinum toxin injections into the flap muscle belly may be used to improve facial symmetry and control hypercontractility [27].

Fascia lata graft harvesting [28] is reserved for adult patients with unilateral facial palsy, where reduced muscle tone and soft tissues ptosis require additional static support. It is not required in children due to the greater elasticity of their soft tissues.

A technical aspect that remains debated in the literature is the most appropriate source of innervation for free gracilis muscle transfer [29,30,31,32]. In our series a formal comparison between neural sources was not possible, since the donor nerve is selected according to the anatomy of the palsy. In patients with bilateral facial palsy, little has changed over the last two decades, and the masseteric nerve remains the most reliable option for flap innervation [33]. However, our long-term follow-up findings suggest that true spontaneity cannot be achieved with masseteric nerve-driven innervation, despite being initially suggested and hypothesized in previous studies [34,35,36]. Automatic, rather than truly spontaneous, activation represents a reasonable compromise in patients who cannot currently achieve a spontaneous smile. Nevertheless, our previous quality-of-life studies have demonstrated that this outcome provides meaningful functional and psychosocial benefits [37,38]; in absence of viable alternative, this approach therefore remains the best available option for innervation in patients with bilateral facial paralysis.

Our philosophy regarding facial animation in patients with unilateral facial palsy has evolved over time. Our guiding principle is that, whenever available, a source of spontaneity should always be provided. In patients with unilateral facial palsy and a healthy, fully functional contralateral facial nerve, facial animation can be reliably achieved through a two-stage surgery, as previously described. The first stage consists of a CFNG using a sural nerve graft to promote axonal regeneration from the healthy contralateral facial nerve to the paralyzed side of the face. Initially, we were concerned about the potential weakening of the healthy side and therefore tended to select the smallest zygomatic branch in order to preserve contralateral facial nerve function. Nowadays, we select a larger-caliber branch of the healthy facial nerve. We have observed that, particularly in children, recovery is consistently complete and no cases of impairment of the donor nerve have been encountered to date. Moreover, even if some degree of reduction in contralateral facial nerve function were to occur, it might contribute to improving facial symmetry after rehabilitation. Careful selection of a zygomatic branch without ocular component is essential, not only to prevent ocular morbidity, but also to ensure appropriate activation during smiling.

The management of facial animation in patients with bilateral incomplete facial palsy has evolved considerably over the years. In these patients, one side of the face retains sufficient residual function and therefore does not require surgical intervention, whereas the contralateral side exhibits severe paralysis and is a candidate for free gracilis muscle transfer. When the donor facial nerve is approached for the cross-facial nerve graft (CFNG) procedure, sacrificing a major-caliber branch is undesirable. Furthermore, the reliability of reinnervation provided by a partially impaired facial nerve remains questionable. To address these challenges, the so-called “supercharged cross-facial nerve graft” has had a significant impact on our practice, modifying our treatment algorithm [12]. This technique combines a partially functional middle branch of the facial nerve with the healthy sided masseteric nerve as dual sources of innervation to the paralyzed side through a sural nerve graft. In our experience, this approach allows restoration of both smile spontaneity and excursion in this challenging subset of patients. By contrast, during the early phase of our experience, these patients underwent gracilis muscle transfer reinnervated predominantly by the ipsilateral masseteric nerve, which reliably restored movement but failed to provide spontaneity and was frequently associated with suboptimal smile synchrony and symmetry. This shift is apparent in the composition of Group B over time (Figure 1): of the seven patients operated up to 2010, five received masseteric innervation and two a cross-face nerve graft, whereas of the twelve operated from 2016 onwards, five received a supercharged cross-face nerve graft, five a cross-face nerve graft and two masseteric innervation, the latter two in patients with severe bilateral involvement in whom the contralateral facial nerve was deliberately spared.

The most relevant development in our practice in recent years concerns the management after flap activation and facial rehabilitation. Based on our long-term experience, we firmly believe that facial rehabilitation is a critical determinant of the final outcome following facial reanimation surgery. Rehabilitation should ideally begin as early as possible, taking into account the patient’s age and ability to cooperate. Whenever feasible, therapy should be initiated before surgery to prepare the patient for facial animation and to facilitate subsequent motor learning. In this process, the facial rehabilitation therapist (in our setting, speech therapist) plays a key role in establishing a trust-based relationship between the healthcare team and the patient’s family, promoting treatment adherence, and ensuring continuity of care. Speech-language therapy should be introduced early in life and maintained across the different stages of the patient’s growth and development. Its goals include supporting oral motor function, swallowing and dysphagia management, as well as speech and language development. Following smile surgery, facial rehabilitation should be reintroduced as soon as muscle activation is detected and continued thereafter to improve muscle strength and enhance motor control, synchronicity, and symmetry of smile [13]. A central component of our rehabilitation protocol is FIT-SAT, which aims to reinforce the cortical motor representation of smiling and thereby increase the spontaneous use of smile in everyday social interactions. Difficulties are related to the purely motor nature of the activation in masseteric nerve-innervated gracilis muscle flap, and there is currently no evidence that spontaneous emotional smiling can be reliably elicited in these patients. Nevertheless, repeated task-specific practice appears to enhance its stability and frequency and promote integration of smiling into daily life activities.

Regarding ancillary periocular procedures, we observed, in agreement with previous literature reports, that only a small number of patients developed lagophthalmos requiring upper eyelid weight implantation and/or lateral tarsal strip surgery. These findings support the well-established concept that patients with congenital facial palsy develop adaptive and compensatory mechanisms for incomplete eyelid closure, which generally provide adequate ocular protection despite the facial deficit [3,39]. Conversely, ocular symptoms and discomfort represent a major source of concern and morbidity in patients with acquired facial palsy and frequently require medical and surgical interventions [39,40].

Several refinements and adjunctive procedures are employed in patients with congenital facial palsy [41]. In our study, 15.6% of patients underwent an upper lip procedure to improve upper lip atrophy. Correction of upper lip atrophy is a specific target of ancillary procedures in these patients, as it may compromise oral competence, impair articulation of certain phonemes, and represents an important esthetic concern [41,42]. In our experience, upper lip volumization is the best strategy available today to reduce upper teeth exposure and improve oral hygiene [43,44]. Fat grafting, performed under general anesthesia from 8 to 10 years of age and under local anesthesia after 16 years, has yielded favorable outcomes; however, several sessions are usually required to achieve an optimal esthetic and functional improvement. Injection of hyaluronic acid or other fillers is certainly another valid solution [45]; however, in our practice it represents a secondary option, as it is not routinely offered within our institutional setting and it is currently available only in private practice. We have also attempted other surgical techniques described in the literature for the correction of upper incisor over-exposure, including mucosal grafting, lip elongation, and Z-plasty. However, in all cases our results were suboptimal, with frequent recurrence.

Botulinum toxin injections have been routinely performed in our practice in patients with facial palsy, with the aim of improving facial symmetry. Botulinum toxin injection into the gracilis muscle belly have been introduced in selected patients with excessive gracilis flap contraction to modulate muscle activation and improve dynamic symmetry [27].

As a maxillofacial surgery unit, we have had the opportunity over the years to observe the craniofacial and dentoskeletal development of patients with congenital facial palsy. Craniofacial skeletal abnormalities, including micrognathia, may be pronounced in patients with bilateral facial palsy and in other syndromic patients with facial paralysis. Indeed, none of the patients with unilateral palsy were treated for facial skeletal malformations. It is noteworthy that, among patients with bilateral facial palsy, only a small proportion (three patients, 5.9%) underwent orthognathic surgery, while a similarly small proportion (two patients, 3.9%) underwent isolated genioplasty. This finding may reflect the psychological and social adaptation of these patients, who, after facial reanimation procedures, may perceive skeletal deformities as less clinically significant. However, this interpretation remains speculative and further studies are needed to investigate this issue. Based on our experience and on patients referred to our center after undergoing orthognathic surgery elsewhere prior to smile reanimation [46], gracilis muscle transfer should ideally precede orthognathic surgery, including adult patients, to avoid soft tissue deformation and stretching that may adversely affect oral competence and lip position. When the timing of smile reanimation is appropriately planned, this complication appears to be preventable.

The final, but no less important, aspect investigated over the years is the long-term stability of the outcomes achieved with facial reanimation using gracilis muscle transfer. As demonstrated by a previous study, no decline in function or deterioration of results over time has been observed. On the contrary, facial movement tends to improve over the years in the majority of patients [47]. In the present series, however, each patient contributed a single postoperative assessment, and durability could therefore not be re-assessed.

**Limitations**. This study has several limitations. It is a retrospective, single-center series, and objective measurements could be obtained only in the 61 of 96 patients with standardized preoperative and postoperative photographs, although these did not differ from the remaining patients in any characteristic examined. Each patient contributed a single postoperative assessment, obtained at a variable point during follow-up, so durability could not be assessed within individual patients. The indication for surgery was established on clinical grounds alone: the objective measurements reported here were obtained retrospectively and were not available at the time of the surgical decision. The intervals between staged procedures are influenced by organizational as much as by clinical factors. Finally, the neural donor is selected according to the anatomy of the palsy rather than independently of the patient, the subgroups are small, and the additional comparisons were exploratory.

## 5. Conclusions

Gracilis neuromuscular transfer has remained the gold standard for facial animation over the years; however, multiple aspects of smile surgery and patient management have evolved substantially. Advances in surgical techniques, the introduction of supercharged cross-face nerve graft, updating in facial retraining protocols, improved understanding of multidisciplinary care, and accumulated experience have shaped the current management of these patients in our institution.

## Figures and Tables

**Table 1:** Characteristics of population.

Characteristic	*n* = 96 (%)
***Sex***	
Male	38 (39.6)
Female	58 (60.4)
***Access pathway***	
Association	58 (60.4)
Other specialists	38 (39.6)
***Age at surgery***	
Total patients (%), mean age (range)	96 (100), 14.8 (5–49)
<18 years (%), mean age	68 (70.8), 8.6
≥18 years (%), mean age	28 (29.2), 30.1
***Facial palsy***	
Unilateral facial palsy	45 (46.9)
Complete	25 (26.1)
Incomplete	20 (20.8)
Bilateral facial palsy	51 (53.1)
Complete	22 (22.9)
Incomplete	29 (30.2)
Isolated	42 (43.8)
Syndromic	54 (56.2)
Moebius	49 (51)
Moebius with Poland	2 (2.1)
Moebius with Turner	1 (1)
CHARGE	2 (2.1)
***Surgery***	
Group A	45 (46.9)
Group B	22 (22.9)
Group C	29 (30.2)
CFNG to gracilis inter-stage interval	
Months (%)	57 (59.4)
Median (IQR)	10 (9–12)
Mean (range)	10.5 (8–17)
Interval between the two gracilis transfers	
Months (%)	29 (30.2)
Median (IQR)	10 (9–11)
Mean (range)	10.3 (6–18)
***Surgical outcome***	
Major complications (%)	2 (2.1)
Minor complications (%)	15 (15.6)
***Follow-up***	
Mean months (median, range)	112.3 (107, 12–241)

CHARGE, coloboma, heart defects, atresia of the choanae, retardation of growth and/or development, genital and/or urinary abnormalities, and ear anomalies and/or deafness. IQR, interquartile range.

**Table 2:** Baseline demographic, clinical and surgical characteristics of the 61 patients included in the objective analysis.

Characteristic	All Included (*n* = 61)	Group A (*n* = 28)	Group B (*n* = 17)	Group C (*n* = 16)
***Demographics***				
Female	38 (62.3)	18 (64.3)	9 (52.9)	11 (68.8)
Male	23 (37.7)	10 (35.7)	8 (47.1)	5 (31.2)
Age at surgery, years, median (range)	8 (5–48)	10 (6–48)	7 (6–25)	7 (5–38)
***Facial palsy***				
Unilateral	28 (45.9)	28 (100.0)	0 (0.0)	0 (0.0)
Complete	18 (29.5)	18 (64.3)	0 (0.0)	0 (0.0)
Incomplete	10 (16.4)	10 (35.7)	0 (0.0)	0 (0.0)
Bilateral	33 (54.1)	0 (0.0)	17 (100.0)	16 (100.0)
Complete	13 (21.3)	0 (0.0)	0 (0.0)	13 (81.2)
Incomplete	20 (32.8)	0 (0.0)	17 (100.0)	3 (18.8)
Isolated	29 (47.5)	25 (89.3)	4 (23.5)	0 (0.0)
Syndromic	32 (52.5)	3 (10.7)	13 (76.5)	16 (100.0)
Moebius syndrome	31 (50.8)	2 (7.1)	13 (76.5)	16 (100.0)
Other syndrome	1 (1.6)	1 (3.6)	0 (0.0)	0 (0.0)
***Surgery***				
Right side operated	21 (34.4)	18 (64.3)	3 (17.6)	0 (0.0)
Left side operated	24 (39.3)	10 (35.7)	14 (82.4)	0 (0.0)
Both sides operated	16 (26.2)	0 (0.0)	0 (0.0)	16 (100.0)
***Source of innervation***				
Cross-facial nerve graft	27 (44.3)	21 (75.0)	6 (35.3)	0 (0.0)
Supercharged cross-facial nerve graft	9 (14.8)	4 (14.3)	5 (29.4)	0 (0.0)
Masseteric nerve	25 (41.0)	3 (10.7)	6 (35.3)	16 (100.0)
Accessory nerve	0 (0.0)	0 (0.0)	0 (0.0)	0 (0.0)
Revision with change in neural source	1 (1.6)	0 (0.0)	1 (5.9)	0 (0.0)
***Follow-up***				
Months, median (range)	102 (12–241)	103 (12–228)	86 (12–241)	146 (12–212)

Values are *n* (%) unless otherwise stated. Group A: unilateral palsy, one side operated. Group B: bilateral palsy, one side operated. Group C: bilateral palsy, both sides operated. Patients are classified according to the source of innervation to which the postoperative measurements refer, that is, the definitive procedure. One patient underwent revision with a change in neural source and is therefore classified under the masseteric nerve, although the index procedure was a cross-facial nerve graft.

**Table 3:** Facial measurements before and after gracilis free-flap reanimation, by study group.

Measure	Before Surgery	After Surgery	Median Change (95% CI)	Improved	*p*
***Symmetry: absolute deviation (mm)***					
Group A (*n* = 28)					
CE deviation, rest	3.9 (2.9–5.2)	3.1 (1.2–4.4)	−0.6 (−1.7 to +0.6)	18/28	0.264
CH deviation, rest	3.9 (2.6–5.1)	1.1 (0.7–3.0)	−2.0 (−3.0 to −1.1)	24/28	<0.0001
CE deviation, smiling	14.7 (9.6–20.6)	2.4 (1.0–4.8)	−11.6 (−14.8 to −8.8)	28/28	<0.0001
CH deviation, smiling	11.8 (8.3–15.2)	3.1 (1.0–4.4)	−8.4 (−10.2 to −6.8)	28/28	<0.0001
Group B (*n* = 17)					
CE deviation, rest	5.2 (2.7–7.6)	3.2 (0.9–5.8)	−1.7 (−4.1 to +1.4)	10/17	0.284
CH deviation, rest	1.6 (1.4–4.0)	2.8 (1.1–3.6)	+0.1 (−1.4 to +1.6)	9/17	0.890
CE deviation, smiling	11.3 (7.3–13.0)	3.0 (1.7–4.4)	−7.4 (−9.9 to −4.1)	15/17	<0.001
CH deviation, smiling	8.3 (4.4–9.9)	3.0 (1.5–4.9)	−4.1 (−6.2 to −1.6)	13/17	0.006
Group C (*n* = 16)					
CE deviation, rest	2.5 (1.6–4.6)	2.7 (1.5–3.8)	−0.6 (−2.4 to +0.9)	9/16	0.404
CH deviation, rest	0.6 (0.5–1.5)	0.8 (0.5–1.4)	−0.1 (−0.7 to +0.4)	8/16	0.597
CE deviation, smiling	2.7 (1.7–7.9)	2.6 (1.1–3.5)	−1.6 (−4.3 to +0.6)	11/16	0.231
CH deviation, smiling	1.2 (0.6–2.5)	1.9 (1.2–3.0)	+0.5 (−0.5 to +1.0)	5/16	0.159
***Commissure excursion of the operated side (mm)***					
Group A (*n* = 28)					
Operated side, rest	20.8 (18.8–22.5)	26.9 (25.5–29.4)	+6.6 (+5.0 to +7.9)	26/28	<0.0001
Operated side, smiling	20.6 (17.2–24.3)	33.5 (30.2–37.4)	+13.5 (+10.6 to +15.8)	27/28	<0.0001
Dynamic excursion	−1.5 (−4.0 to 2.1)	6.0 (3.4–9.1)	+6.8 (+4.6 to +9.1)	25/28	<0.0001
Group B (*n* = 17)					
Operated side, rest	20.8 (19.0–23.0)	27.7 (26.7–29.9)	+6.7 (+4.7 to +9.0)	17/17	<0.0001
Operated side, smiling	20.2 (18.1–23.5)	32.3 (30.3–34.6)	+11.6 (+9.0 to +14.0)	17/17	<0.0001
Dynamic excursion	−0.1 (−1.1 to 1.8)	3.5 (1.9–8.2)	+5.0 (+2.1 to +7.3)	15/17	0.006
Group C (*n* = 16)					
Operated side, rest	21.9 (20.7–23.6)	27.2 (23.6–27.9)	+3.9 (+1.9 to +5.8)	16/16	<0.0001
Operated side, smiling	22.4 (20.8–23.2)	31.1 (29.5–34.0)	+9.1 (+6.5 to +11.5)	16/16	<0.0001
Dynamic excursion	0.2 (−0.8 to 0.8)	5.2 (3.7–6.7)	+4.8 (+3.3 to +6.8)	16/16	<0.0001
All patients (*n* = 61)					
Operated side, rest	20.9 (19.6–23.1)	27.3 (25.4–29.1)	+5.9 (+4.8 to +6.8)	59/61	<0.0001
Operated side, smiling	20.9 (19.3–23.8)	32.3 (30.0–35.9)	+11.6 (+10.2 to +13.1)	60/61	<0.0001
Dynamic excursion	−0.2 (−2.1 to 1.8)	5.5 (2.6–8.1)	+5.6 (+4.5 to +7.0)	56/61	<0.0001

Values are median (interquartile range) in millimeters. Change is the Hodges–Lehmann estimate of the median within-patient difference (postoperative minus preoperative), with its 95% confidence interval. CE, commissure excursion; CH, commissure height; CI, confidence interval. Group A, unilateral palsy with one side operated; Group B, bilateral palsy with one side operated; Group C, bilateral palsy with both sides operated. Deviation is the absolute right-to-left difference; lower values indicate greater symmetry. Dynamic excursion is the commissure position on smiling minus the position at rest on the same side and is a signed quantity. In patients with both sides operated, the operated-side value is the mean of the two sides. “Improved” counts a decrease for deviation measures and an increase for excursion measures. *p* values are from the exact Wilcoxon signed-rank test, adjusted with the Holm–Bonferroni procedure within each block and group; where all patients changed in the same direction the exact test attains its lower bound and the value is reported as *p* < 0.0001.

## Data Availability

The original contributions presented in this study are included in the article/Supplementary Material. Further inquiries can be directed to the corresponding author).

## Supplementary Materials

The following supporting information can be downloaded at https://www.mdpi.com/article/10.3390/medicina62102039/s1, Figure S1: Internal control, measurements of the non-operated side; Figure S2: Effect of age at surgery on commissure excursion deviation; Figure S3: Outcome according to the amount of gracilis harvested. Table S1: Comparison between the 61 patients included in the objective analysis and the 35 patients excluded because standardized photographs were not available.; Table S2: Internal control: measurements of the non-operated side before and after surgery, in the 45 patients in whom a single side was reanimated.; Table S3: Descriptive statistics of all measurements before and after surgery; Table S4: Paired comparisons between preoperative and postoperative measurements; Table S5: Postoperative commissure excursion measurements according to the neural source used for reinnervation, within each study group.
