# Supplementary material for: Surgical Treatment of Congenital Facial Palsy: A Retrospective Analysis of More than 20 Years’ Experience in Smile Surgery
Source: Medicina (Kaunas). 2026 Oct 21;62(10):2039. doi: 10.3390/medicina62102039 (PMC13707142; doi:10.3390/medicina62102039)
Supplement: Supplementary file 1 [file medicina-62-02039-s001.zip › medicina-4431206-supplementary.pdf]

Supplementary Materials

Surgical treatment of congenital facial palsy: a retrospective analysis of more than 20 years' experience in smile surgery.

Andrea Ferri[1]; Giorgia Menapace[1*]; Cecilia Zannoni[1]; Laura Salvatori[1]; Laganà Francesco[2]; Silvano Ferrari[1]; Bernardo Bianchi[2]

Table S1. Comparison between the 61 patients included in the objective analysis and the 35 patients excluded because standardized photographs were not available.

Characteristic	Included (n = 61)	Excluded (n = 35)	p	Test
Demographics				
Female	38 (62.3)	20 (57.1)	0.619	Chi-square
Age at surgery, years, median (range)	8 (5–48)	12 (6–49)	0.062	Mann–Whitney U
Facial palsy				
Bilateral palsy	33 (54.1)	18 (51.4)	0.801	Chi-square
Complete palsy	31 (50.8)	16 (45.7)	0.630	Chi-square
Syndromic aetiology	32 (52.5)	22 (62.9)	0.323	Chi-square
Moebius syndrome	31 (50.8)	21 (60.0)	0.385	Chi-square
Surgery				
Year of surgery, median (range)	2016 (2003–2024)	2014 (2005–2025)	0.512	Mann–Whitney U
Both sides operated	16 (26.2)	13 (37.1)	0.262	Chi-square
Source of innervation				
Cross-facial nerve graft	28 (45.9)	19 (54.3)	0.429	Chi-square
Supercharged cross-facial nerve graft	9 (14.8)	1 (2.9)	0.087	Fisher's exact
Masseteric nerve	25 (41.0)	15 (42.9)	0.858	Chi-square
Accessory nerve	0 (0.0)	1 (2.9)	0.365	Fisher's exact
Follow-up				
Follow-up, months, median (range)	102 (12–241)	110 (12–219)	0.743	Mann–Whitney U

Values are n (%) unless otherwise stated. All tests two-sided. These comparisons were descriptive and no correction for multiple testing was applied.

Table S2. Internal control: measurements of the non-operated side before and after surgery, in the 45 patients in whom a single side was reanimated.

Measure	Before surgery	After surgery	Median change (95% CI)	p
**Group A (n = 28)**				
Non-operated side, rest	23.7 (22.4–25.5)	23.6 (22.1–27.0)	+0.2 (−1.1 to +1.4)	0.831

Measure	Before surgery	After surgery	Median change (95% CI)	p
Non-operated side, smiling	36.3 (31.1–39.3)	34.0 (30.7–37.6)	−0.9 (−3.4 to +0.7)	0.327
Dynamic excursion, non-operated side	11.6 (8.3–15.0)	10.5 (9.4–11.6)	−1.5 (−3.4 to +0.5)	0.115
**Group B (n = 17)**				
Non-operated side, rest	23.6 (22.5–25.5)	24.5 (23.1–28.0)	+1.3 (−0.2 to +3.8)	0.089
Non-operated side, smiling	30.3 (25.9–34.5)	32.7 (30.0–35.5)	+0.4 (−0.9 to +3.9)	0.517
Dynamic excursion, non-operated side	5.7 (4.5–11.0)	6.9 (4.4–8.4)	−0.8 (−2.7 to +0.8)	0.329

Values are median (interquartile range) in millimetres. Change is the Hodges–Lehmann estimate of the median within-patient difference. p values from the exact Wilcoxon signed-rank test. No measure changed significantly.

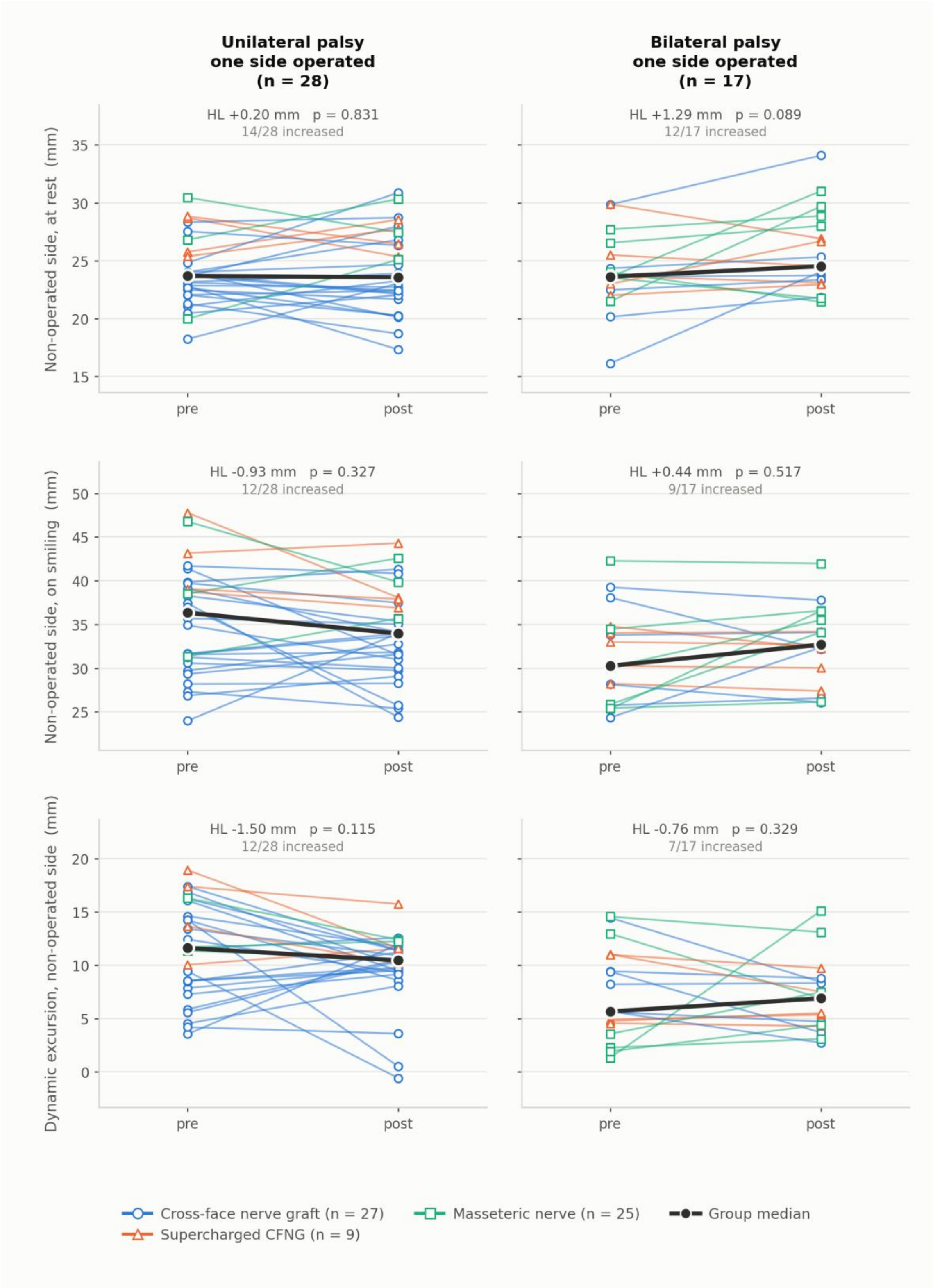

Figure S1. Internal control: measurements of the non-operated side in the 45 patients in whom a single side was reanimated. No measure changed significantly between the preoperative and the postoperative assessment, supporting the view that the changes observed on the operated side reflect a true surgical effect rather than differences in photographic technique or measurement scale.

Table S3. Descriptive statistics of all measurements before and after surgery.

Measure	n	Pre median	Pre IQR	Pre mean	Pre SD	Post median	Post IQR	Post mean	Post SD
Symmetry (absolute right-to-left deviation)									
**Group A (n = 28)**									
CE deviation, rest	28	3.9	2.9–5.2	4.1	2.1	3.1	1.2–4.4	3.7	3.2
CH deviation, rest	28	3.9	2.6–5.1	3.8	1.9	1.1	0.7–3.0	1.7	1.4
CE deviation, smiling	28	14.7	9.6–20.6	15.4	7.3	2.4	1.0–4.8	3.5	3.3
CH deviation, smiling	28	11.8	8.3–15.2	12.0	4.1	3.1	1.0–4.4	3.4	3.2
**Group B (n = 17)**									
CE deviation, rest	17	5.2	2.7–7.6	5.2	2.7	3.2	0.9–5.8	3.8	3.3
CH deviation, rest	17	1.6	1.4–4.0	2.7	2.3	2.8	1.1–3.6	2.9	2.4
CE deviation, smiling	17	11.3	7.3–13.0	10.7	5.6	3.0	1.7–4.4	3.8	3.3
CH deviation, smiling	17	8.3	4.4–9.9	7.4	3.6	3.0	1.5–4.9	3.6	2.8
**Group C (n = 16)**									
CE deviation, rest	16	2.5	1.6–4.6	3.7	2.9	2.7	1.5–3.8	2.8	2.0
CH deviation, rest	16	0.6	0.5–1.5	1.1	1.0	0.8	0.5–1.4	1.1	1.3
CE deviation, smiling	16	2.7	1.7–7.9	4.9	5.4	2.6	1.1–3.5	2.8	1.9
CH deviation, smiling	16	1.2	0.6–2.5	2.5	4.0	1.9	1.2–3.0	2.1	1.4
Commissure excursion, operated side									
**Group A (n = 28)**									
Operated side, rest	28	20.8	18.8–22.5	20.8	2.9	26.9	25.5–29.4	27.1	3.2
Operated side, smiling	28	20.6	17.2–24.3	20.0	5.5	33.5	30.2–37.4	33.4	5.3
Dynamic excursion	28	−1.5	−4.0 to 2.1	−0.8	4.8	6.0	3.4–9.1	6.3	4.2
**Group B (n = 17)**									
Operated side, rest	17	20.8	19.0–23.0	21.1	3.8	27.7	26.7–29.9	28.1	3.6
Operated side, smiling	17	20.2	18.1–23.5	20.6	4.2	32.3	30.3–34.6	32.1	4.0
Dynamic excursion	17	−0.1	−1.1 to 1.8	−0.5	5.0	3.5	1.9–8.2	4.0	5.3
**Group C (n = 16)**									
Operated side, rest	16	21.9	20.7–23.6	22.3	2.5	27.2	23.6–27.9	26.2	2.7

Measure	n	Pre median	Pre IQR	Pre mean	Pre SD	Post median	Post IQR	Post mean	Post SD
Operated side, smiling	16	22.4	20.8–23.2	22.6	3.3	31.1	29.5–34.0	31.9	3.5
Dynamic excursion	16	0.2	−0.8 to 0.8	0.3	2.2	5.2	3.7–6.7	5.6	3.6
**All patients (n = 61)**									
Operated side, rest	61	20.9	19.6–23.1	21.3	3.1	27.3	25.4–29.1	27.2	3.2
Operated side, smiling	61	20.9	19.3–23.8	20.9	4.7	32.3	30.0–35.9	32.6	4.5
Dynamic excursion	61	−0.2	−2.1 to 1.8	−0.4	4.3	5.5	2.6–8.1	5.5	4.4
Internal control, non-operated side									
**Group A (n = 28)**									
Non-operated side, rest	28	23.7	22.4–25.5	24.0	2.9	23.6	22.1–27.0	24.2	3.5
Non-operated side, smiling	28	36.3	31.1–39.3	35.4	6.1	34.0	30.7–37.6	34.0	5.2
Dynamic excursion, non-operated side	28	11.6	8.3–15.0	11.4	4.5	10.5	9.4–11.6	9.8	3.4
**Group B (n = 17)**									
Non-operated side, rest	17	23.6	22.5–25.5	24.0	3.4	24.5	23.1–28.0	25.7	3.6
Non-operated side, smiling	17	30.3	25.9–34.5	31.4	5.4	32.7	30.0–35.5	32.7	4.4
Dynamic excursion, non-operated side	17	5.7	4.5–11.0	7.4	4.4	6.9	4.4–8.4	7.0	3.4

All values in millimetres. IQR, interquartile range; SD, standard deviation.

Table S4. Paired comparisons between preoperative and postoperative measurements.

Measure	n	HL	95% CI low	95% CI high	Wilcoxon p	Holm p	rank-biserial	Improve d	Shapiro–Wilk p	Paired t p
Symmetry (absolute right-to-left deviation)										
**Group A (n = 28)**										
CE deviation, rest	28	−0.6	−1.7	+0.6	0.264	0.264	−0.25	18/28	0.032	0.630
CH deviation, rest	28	−2.0	−3.0	−1.1	< 0.0001	< 0.001	−0.81	24/28	0.904	< 0.0001
CE deviation, smiling	28	−11.6	−14.8	−8.8	< 0.0001	< 0.0001	−1.00	28/28	0.127	< 0.0001
CH deviation, smiling	28	−8.4	−10.2	−6.8	< 0.0001	< 0.0001	−1.00	28/28	0.656	< 0.0001
**Group B (n = 17)**										

Measure	n	HL	95% CI low	95% CI high	Wilcoxon p	Holm p	rank-biserial	Improved	Shapiro–Wilk p	Paired t p
CE deviation, rest	17	−1.7	−4.1	+1.4	0.284	0.568	−0.31	10/17	0.308	0.283
CH deviation, rest	17	+0.1	−1.4	+1.6	0.890	0.890	+0.05	9/17	0.465	0.827
CE deviation, smiling	17	−7.4	−9.9	−4.1	< 0.001	0.002	−0.90	15/17	0.140	< 0.001
CH deviation, smiling	17	−4.1	−6.2	−1.6	0.006	0.017	−0.74	13/17	0.699	0.003
**Group C (n = 16)**										
CE deviation, rest	16	−0.6	−2.4	+0.9	0.404	0.807	−0.25	9/16	0.187	0.265
CH deviation, rest	16	−0.1	−0.7	+0.4	0.597	0.807	−0.16	8/16	0.008	0.949
CE deviation, smiling	16	−1.6	−4.3	+0.6	0.231	0.694	−0.35	11/16	0.040	0.108
CH deviation, smiling	16	+0.5	−0.5	+1.0	0.159	0.636	+0.41	5/16	< 0.0001	0.684
Commissure excursion, operated side										
**Group A (n = 28)**										
Operated side, rest	28	+6.6	+5.0	+7.9	< 0.0001	< 0.0001	+0.99	26/28	0.627	< 0.0001
Operated side, smiling	28	+13.5	+10.6	+15.8	< 0.0001	< 0.0001	+1.00	27/28	0.253	< 0.0001
Dynamic excursion	28	+6.8	+4.6	+9.1	< 0.0001	< 0.0001	+0.92	25/28	0.541	< 0.0001
**Group B (n = 17)**										
Operated side, rest	17	+6.7	+4.7	+9.0	< 0.0001	< 0.0001	+1.00	17/17	0.701	< 0.0001
Operated side, smiling	17	+11.6	+9.0	+14.0	< 0.0001	< 0.0001	+1.00	17/17	0.918	< 0.0001
Dynamic excursion	17	+5.0	+2.1	+7.3	0.006	0.006	+0.74	15/17	0.420	0.003
**Group C (n = 16)**										
Operated side, rest	16	+3.9	+1.9	+5.8	< 0.0001	< 0.0001	+1.00	16/16	0.095	< 0.001
Operated side, smiling	16	+9.1	+6.5	+11.5	< 0.0001	< 0.0001	+1.00	16/16	0.895	< 0.0001
Dynamic excursion	16	+4.8	+3.3	+6.8	< 0.0001	< 0.0001	+1.00	16/16	0.010	< 0.0001
**All patients (n = 61)**										
Operated side, rest	61	+5.9	+4.8	+6.8	< 0.0001	< 0.0001	+0.99	59/61	0.573	< 0.0001
Operated side, smiling	61	+11.6	+10.2	+13.1	< 0.0001	< 0.0001	+1.00	60/61	0.094	< 0.0001
Dynamic excursion	61	+5.6	+4.5	+7.0	< 0.0001	< 0.0001	+0.89	56/61	0.064	< 0.0001
Internal control, non-operated side										
**Group A (n = 28)**										

Measure	n	HL	95% CI low	95% CI high	Wilcoxon p	Holm p	rank-biserial	Improved	Shapiro–Wilk p	Paired t p
Non-operated side, rest	28	+0.2	−1.1	+1.4	0.831	0.831	+0.05	14/28	0.786	0.673
Non-operated side, smiling	28	−0.9	−3.4	+0.7	0.327	0.654	−0.22	12/28	0.156	0.151
Dynamic excursion, non-operated side	28	−1.5	−3.4	+0.5	0.115	0.344	−0.34	12/28	0.951	0.083
**Group B (n = 17)**										
Non-operated side, rest	17	+1.3	−0.2	+3.8	0.089	0.266	+0.48	12/17	0.087	0.054
Non-operated side, smiling	17	+0.4	−0.9	+3.9	0.517	0.658	+0.19	9/17	0.052	0.217
Dynamic excursion, non-operated side	17	−0.8	−2.7	+0.8	0.329	0.658	−0.28	7/17	0.008	0.738

HL, Hodges–Lehmann estimate of the median within-patient difference (postoperative minus preoperative), in millimetres, with its 95% confidence interval derived from the Wilcoxon distribution. Wilcoxon p from the exact signed-rank test; Holm p, the same value after Holm–Bonferroni adjustment within each block of measures and each group. Rank-biserial is the matched-pairs effect size. Shapiro–Wilk p refers to the distribution of the within-patient differences; the paired t-test is reported as a confirmatory analysis. "Improved" counts a decrease for deviation measures and an increase for excursion measures. Where all patients changed in the same direction the exact test attains its lower bound and the value is reported as $p < 0.0001$.

Definitions and statistical methods

Groups. Group A, unilateral palsy with one side operated; Group B, bilateral palsy with one side operated; Group C, bilateral palsy with both sides operated. The stratification was adopted because the right-to-left deviation has a different meaning depending on whether one or both sides are affected and reanimated.

CE deviation / CH deviation. Absolute right-to-left difference in commissure excursion and commissure height. Lower values indicate greater symmetry.

Operated side. Commissure excursion of the operated side. In the 16 patients with both sides operated this is the mean of the two sides.

Dynamic excursion. Commissure position on smiling minus position at rest on the same side. It is a signed quantity and is negative before surgery in 33 of the 61 patients, because on smiling the paretic commissure is displaced towards the contralateral side.

Statistical methods. Exact Wilcoxon signed-rank test for paired data, two-sided; Hodges–Lehmann estimator of the median within-patient change with 95% confidence interval; matched-pairs rank-biserial correlation as effect size; Holm–Bonferroni correction within each block of measures and each group. Analyses performed in Python 3.11 using SciPy 1.17, statsmodels 0.15 and pandas 3.0.

Table S5. Postoperative commissure excursion measurements according to the neural source used for reinnervation, within each study group. Values are median (interquartile range) in millimetres. Subgroups are reported descriptively: they are too small for a formal comparison, and the choice of the neural donor is determined by the anatomy of the palsy rather than assigned independently of the patient, so differences between neural sources are confounded with the indication for their use.

Neural source	n	CE on smiling	CE deviation on smiling	Dynamic excursion	Change in dynamic excursion
Group A — unilateral palsy, one side reanimated (n = 28)					
Cross-face nerve graft	21	31.1 (29.1–36.1)	1.8 (0.8–4.6)	5.9 (2.4–9.0)	5.6 (1.7–9.2)
Supercharged cross-face nerve graft	4	35.6 (33.1–37.8)	3.1 (2.0–6.4)	5.5 (4.2–6.7)	7.9 (6.3–9.3)
Masseteric nerve	3	35.9 (35.5–37.2)	4.0 (3.4–5.7)	8.9 (8.4–10.1)	13.3 (12.6–14.6)
Group B — bilateral palsy, one side reanimated (n = 17)					
Cross-face nerve graft	6	31.3 (30.1–33.8)	2.5 (1.8–3.7)	2.3 (1.8–3.3)	3.2 (1.9–6.3)
Supercharged cross-face nerve graft	5	32.7 (30.7–34.6)	2.7 (1.6–3.0)	3.7 (2.6–8.2)	2.8 (2.7–7.7)
Masseteric nerve	6	32.2 (27.3–35.9)	4.9 (2.8–9.1)	7.1 (−1.3 to 9.5)	6.6 (5.1–10.0)
Group C — bilateral palsy, both sides reanimated (n = 16)					
Masseteric nerve	16	31.1 (29.5–34.0)	2.6 (1.1–3.5)	5.2 (3.7–6.7)	4.2 (2.9–6.7)

CE, commissure excursion. Dynamic excursion is the position of the commissure on smiling minus its position at rest on the same side, and is a signed quantity. Change in dynamic excursion is the postoperative minus the preoperative value.

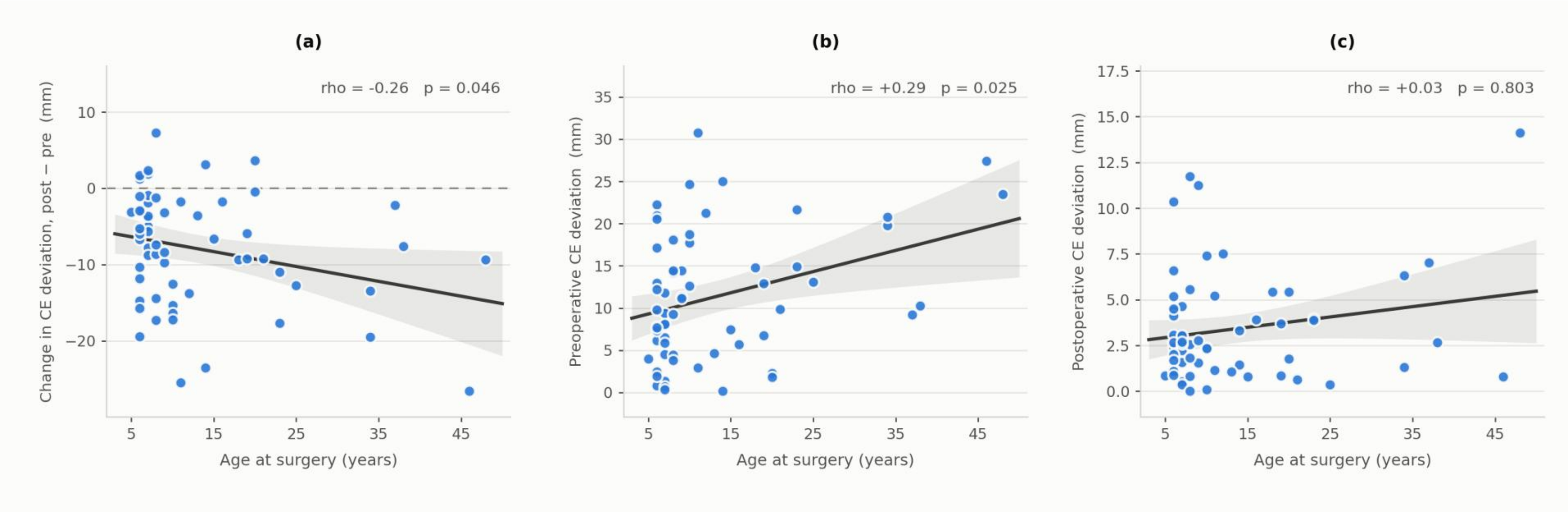

Figure S2. Effect of age at surgery on commissure excursion deviation during smiling in the 61 patients analyzed. (**a**) The within-patient change is larger in older patients; (**b**) older patients presented with greater preoperative asymmetry; (**c**) the postoperative value is unrelated to age. rho, Spearman rank correlation coefficient; the line is a linear regression with its 95% confidence band. The apparent association in (**a**) reflects the dependence of the change on the baseline value (rho = −0.93) rather than an effect of age on the result achieved.

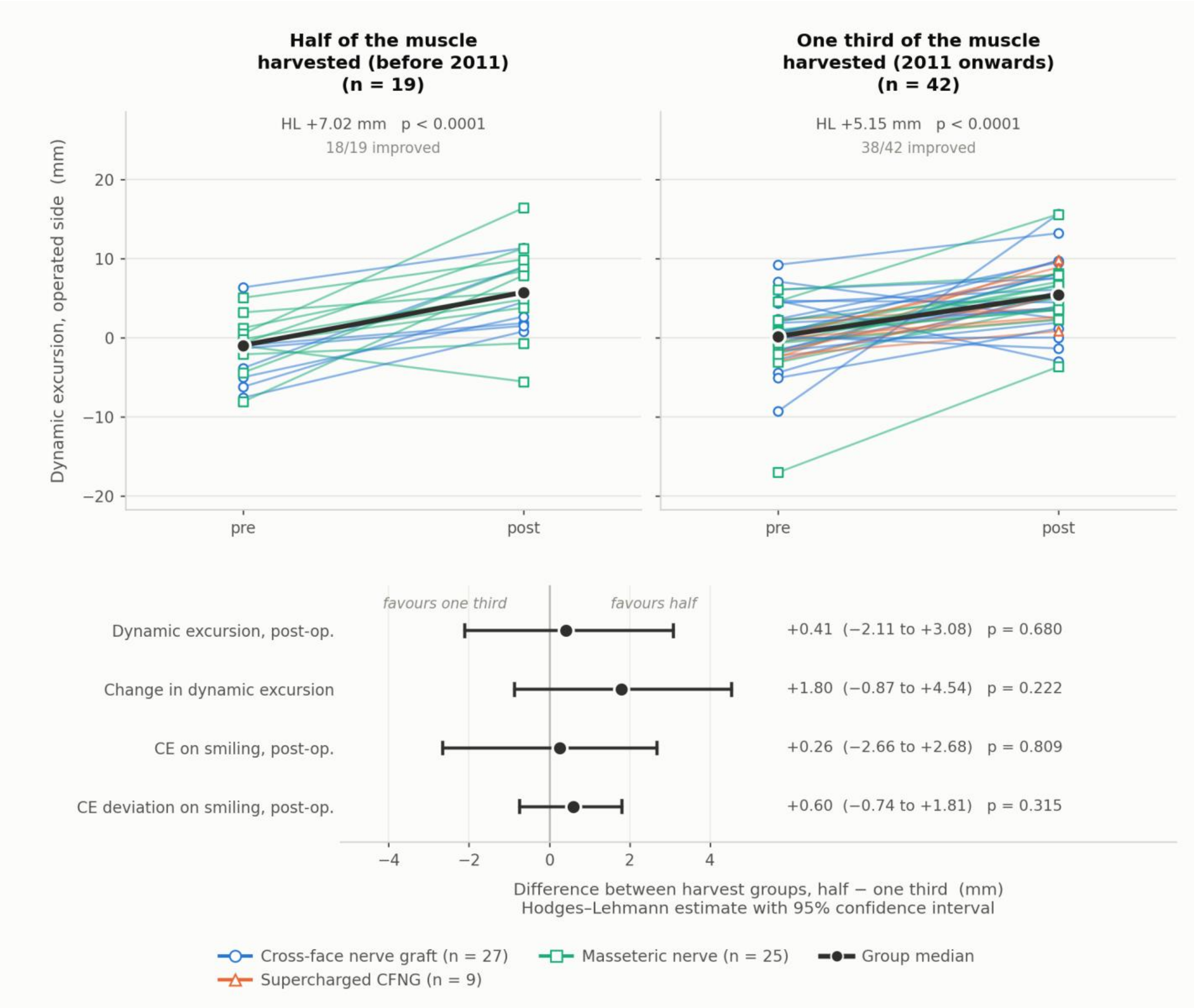

Figure S3. Outcome according to the amount of gracilis harvested. Since 2011 the harvest was reduced from one half to one third of the muscle; patients are therefore grouped as operated before 2011 (half, n = 19) or from 2011 onwards (one third, n = 42). **Top panels:** dynamic excursion of the operated side before and after reanimation. Each line is one patient, coloured by

the neural source driving the flap; the thick black line joins the group medians. Dynamic excursion is a signed quantity, defined as the position of the commissure on smiling minus its position at rest on the same side, so negative values indicate a commissure that moves inwards on smiling. HL = Hodges–Lehmann estimate of the median within-patient change; *p* from the exact Wilcoxon signed-rank test. **Bottom panel:** between-group differences (half minus one third) for four post-operative outcomes, expressed as Hodges–Lehmann estimates with 95% confidence intervals; *p* from the Mann–Whitney *U* test. All intervals include zero. CE = commissure excursion. Harvest size is defined by a calendar threshold and is therefore confounded with surgical era, follow-up duration and the type of neural input available at the time (supercharged CFNG was introduced in 2016); the comparison should be read as a between-era comparison rather than as an isolated effect of muscle volume.
